# Hyperphosphorylation of Human Osteopontin and Its Impact on Structural Dynamics and Molecular Recognition

**DOI:** 10.1021/acs.biochem.1c00050

**Published:** 2021-04-20

**Authors:** Borja Mateos, Julian Holzinger, Clara Conrad-Billroth, Gerald Platzer, Szymon Żerko, Marco Sealey-Cardona, Dorothea Anrather, Wiktor Koźmiński, Robert Konrat

**Affiliations:** † Department of Structural and Computational Biology, University of Vienna, Max Perutz Labs, Vienna BioCenter Campus 5, 1030 Vienna, Austria; ‡ Faculty of Chemistry, Biological and Chemical Research Centre, University of Warsaw, 02093 Warsaw, Poland; § CALYXHA Biotechnologies GmbH, Karl-Farkas-Gasse 22, 1030 Vienna, Austria; ∥ Mass Spectrometry Facility, Max Perutz Laboratories, Vienna BioCenter Campus 5, Dr. Bohr Gasse 3, 1030 Vienna, Austria

## Abstract

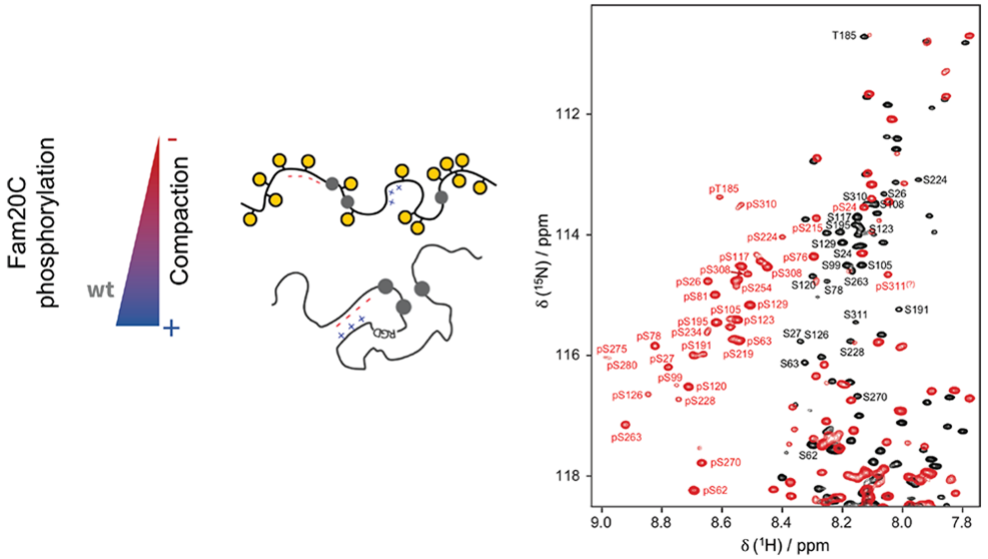

Protein phosphorylation is an abundant post-translational modification (PTM) and an essential modulator of protein functionality in living cells. Intrinsically disordered proteins (IDPs) are particular targets of PTM protein kinases due to their involvement in fundamental protein interaction networks. Despite their dynamic nature, IDPs are far from having random-coil conformations but exhibit significant structural heterogeneity. Changes in the molecular environment, most prominently in the form of PTM via phosphorylation, can modulate these structural features. Therefore, how phosphorylation events can alter conformational ensembles of IDPs and their interactions with binding partners is of great interest. Here we study the effects of hyperphosphorylation on the IDP osteopontin (OPN), an extracellular target of the Fam20C kinase. We report a full characterization of the phosphorylation sites of OPN using a combined nuclear magnetic resonance/mass spectrometry approach and provide evidence for an increase in the local flexibility of highly phosphorylated regions and the ensuing overall structural elongation. Our study emphasizes the simultaneous importance of electrostatic and hydrophobic interactions in the formation of compact substates in IDPs and their relevance for molecular recognition events.

Protein phosphorylation is an abundant post-translational modification that adds an extra layer of complexity to the regulation of cellular fate, particularly in intrinsically disordered proteins because of their inherent accessibility.^1^ Regulation of cellular signaling by phosphorylation is associated with conformational changes^2−7^ and modulation of binding events^8,9^ and, recently, has been linked to the formation of membraneless organelles.^10^

The extracellular matrix (ECM) contains a large fraction of phosphorylated proteins, and many of them have been observed in breast and lung cancer samples.^11−13^ Among these ECM proteins, osteopontin (OPN) and caseins have the highest fractions of potential phosphorylation sites.^12^ OPN, also known as secreted phosphoprotein 1 (SPP1), is a secreted extracellular protein that exerts its functionality by binding to integrin and CD44 receptors and is reported to be implicated in apoptosis, wound healing, inflammation, tumor growth, tumor progression, and tumor metastasis.^14−16^ It is tightly regulated by glycosylation, phosphorylation and cleavage,^17,18^ and is secreted in its unphosphorylated^19^ or phosphorylated^20^ form. Human OPN is mainly phosphorylated by Fam20C (67% of the reported phosphorylated sites).^21,22^ Fam20C kinase is located in the Golgi lumen and responsible for most of the phosphorylation in the ECM. It recognizes primarily a S-x-E/pS motif but also shows a certain promiscuity with respect to other amino acid motifs (e.g., T-x-E or S-x-D).^21^ OPN contains 28 potential Fam20C specific motifs, causing ≤14% of the residues being phosphorylated. The degree of OPN phosphorylation has been associated with Raine syndrome, a rare disease characterized by generalized osteosclerosis with periosteal bone formation, characteristic facial dysmorphism, brain abnormalities, including intracerebral calcifications, and in some cases neonatal death.^13,23,24^ Its abnormal phosphorylation patterns are directly connected to Fam20C mutations. Furthermore, the phosphorylation of OPN regulates its binding interaction with hydroxyapatite and hence the formation and growth of the mineral phase in bone material,^25−31^ as well as bone remodeling and calcification.^13,28,32,33^ On top of that, ECM phosphoproteome homeostatis, in particular OPN phosphorylation, has been associated with tumor cell progression,^34^ macrophage migration,^35^ and host–cell interactions.^36^

Nuclear magnetic resonance (NMR) spectroscopy has matured into an exquisite tool to tackle PTMs and to study the structural dynamics of the protein of interest under nativelike conditions.^37−44^ Although denaturing conditions have been particularly useful for characterizing modified sites,^43,44^ it is important to take into account the fact that the conformational ensembles of IDPs are drastically affected by the presence of denaturing agents, as IDPs are far from being merely unfolded.^45^ With respect to OPN, several important features that account for the modulation of compaction, binding and function of the unphosphorylated form have been identified.^46−48^ Here, we present an NMR-based strategy for structurally characterizing the fully phosphorylated protein and the dynamics of the hyperphosphorylated OPN. For this purpose, a stable HEK293T cell line expressing Fam20C was used to obtain the pure functional kinase.^21^ The degree of phosphorylation and the homogeneity of the modified phospho-residue patterns were optimized in a controlled *in vitro* reaction. NMR signal assignment experiments reveal a downfield shift of a majority of the serine and individual threonine ^1^H^N^ NMR signals due to intraresidue hydrogen bonding between the phosphate and backbone amide groups in unstructured regions.^49^ The experimental NMR data set is complemented by a mass spectrometry (MS) analysis. The putative biological relevance of these findings is outlined with studies of the interaction with heparin and hyaluronic acid, which are present in proteoglycans and the ECM, and the comparison of our results to reported affinities for integrin receptors, natural binders of OPN.

## Results

### NMR/MS-Based Phosphoprofiling of OPN

**Figure 1:**
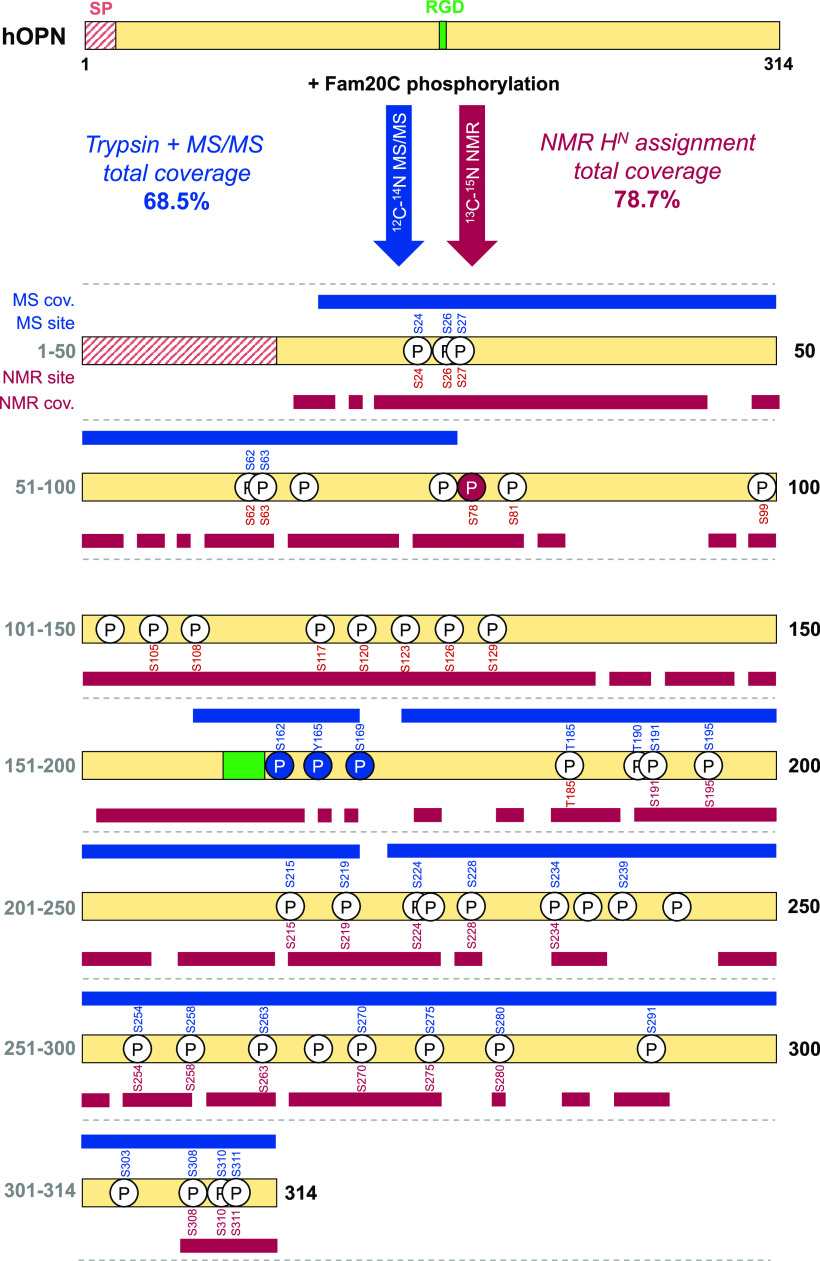
Scheme of OPN residues phosphorylated *in vitro* by Fam20C, identified by MS and NMR spectroscopy. White circles represent previously identified phosphorylation sites.^21,22^ Blue and red circles indicate the phosphorylation sites newly identified by MS and NMR spectroscopy, respectively. The blue and red bars indicate the coverage of MS and H^N^ NMR assignments, respectively.

A highly pure unphosphorylated ^13^C/^15^N *Homo sapiens* OPN was expressed recombinantly in *Escherichia coli* (Figures S1 and S2). The functional wild type (wt) and D478A (kinase-dead mutant) Fam20C kinases were expressed in HEK293T stable expression cells (Figure S3). The *in vitro* phosphorylation reaction of OPN was optimized from previously reported conditions^13^ (see the Supporting Information for detailed method protocols). A combined approach using MS and NMR spectroscopy was carried out for the identification of the phosphorylation sites. The results are summarized in Figure 1. The total sequence coverage of the MS/MS experiments is 68.5%, and 28 phosphorylation events are identified (see Figure S4). Among them, 17 of the 22 canonical motifs are found to be phosphorylated (S24, S26, S27, S62, S63, S195, S224, S234, S254, S263, S270, S275, S280, S291, S303, S308, and S310). A plausible alternative motif (T-x-E) is also found to be phosphorylated in position T185. Other phosphorylated residues do not follow the mentioned motif, although some of them were found to be phosphorylated in mammalian cells^21^ or bodily fluids (milk)^22^ (Figure S5), suggesting a certain promiscuity of the kinase and/or the activity of other unreported kinases.^21^

**Figure 2:**
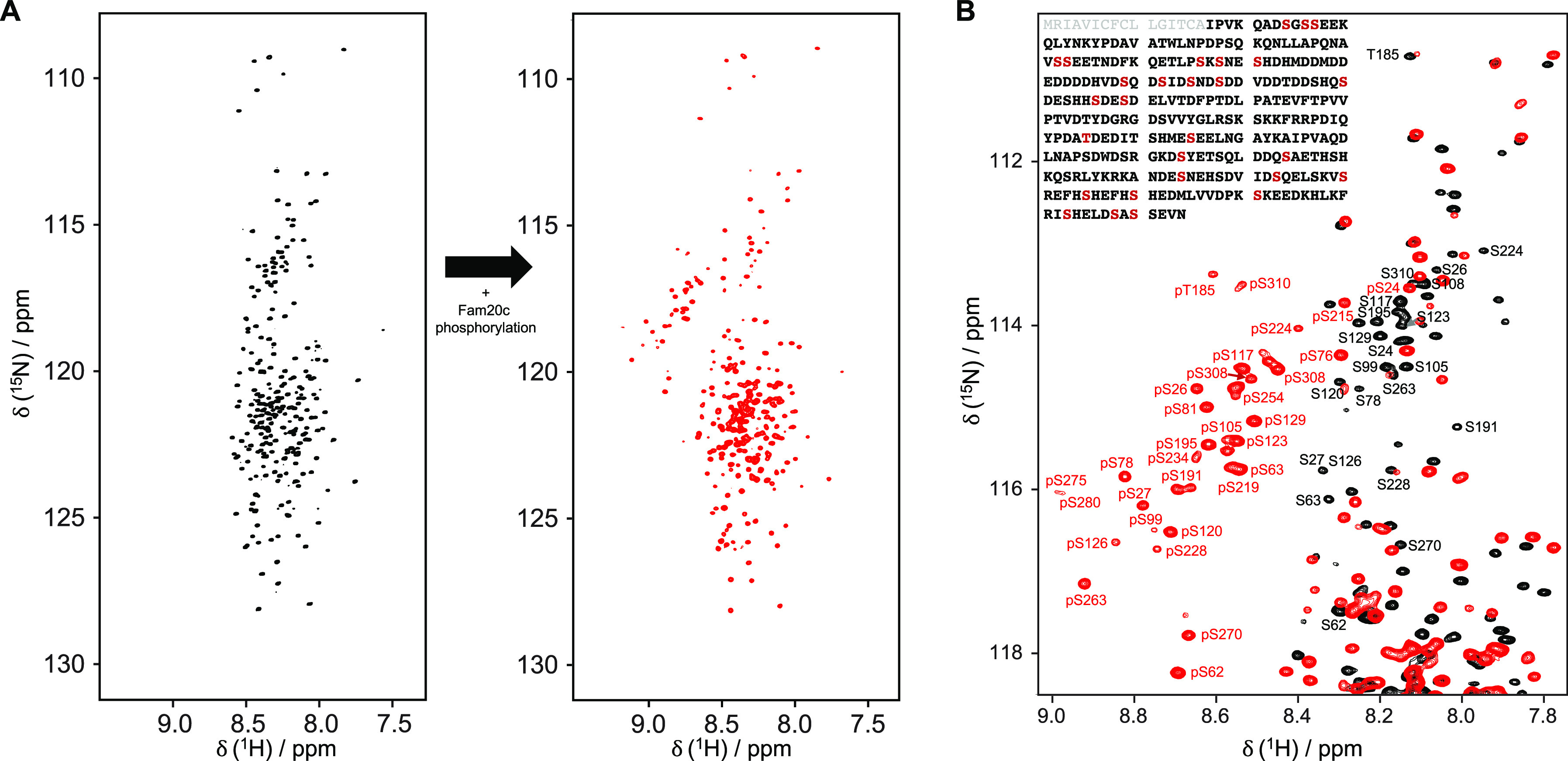
NMR fingerprint of OPN hyperphosphorylation. (A) ^1^H–^15^N HSQC NMR spectra of OPN before (black) and after (red) phosphorylation by Fam20C. Note how many serine residues [δ (^15^N) ≈116 ppm] experience a downfield shift in the ^1^H dimension. (B) Close-up of the serine region of ^1^H–^15^N HSQC NMR spectra of OPN before (black) and after (red) phosphorylation by Fam20C. The protein sequence with the S-x-E/pS sites colored red is shown in the top left corner. The signal peptide, which is not present in our construct, is colored gray.

Among those (e.g., S162, Y165, and S169), previously unreported sites are reliably identified by MS on peptides GDSVVYGLR and GDSVVYGLRSK. The fragment pattern is continuous and shows the properties of phospho spectra. Resonance assignment by NMR spectroscopy was achieved for 78.7% of all H^N^ signals (deposited in the BMRB^50^ as entry 50447 and Table S1). Twenty-eight phosphorylation events are identified on the basis of the ^1^H^N^ downfield shifts (Figure 2). Twenty of the 22 canonical phosphorylation motifs are found to be phosphorylated (S24, S26, S27, S62, S63, S78, S81, S120, S126, S129, S195, S224, S234, S254, S263, S270, S275, S280, S308, and S310), and four noncanonical but plausible phosphorylated motifs (T185, T-x-E; and S99, S105, and S108, S-x-D), previously also found in phosphorylated OPN extracted from milk.^22^ The four remaining phosphorylated residues display a noncanonical phosphorylation motif. Some of these noncanonical phosphorylations are identified both by MS phosphomapping and NMR assignment [S191, S215, S228, and S258 (see Figure 1 and Figure S5)].

### Phosphorylation Increases Local Flexibility in OPN

**Figure 3:**
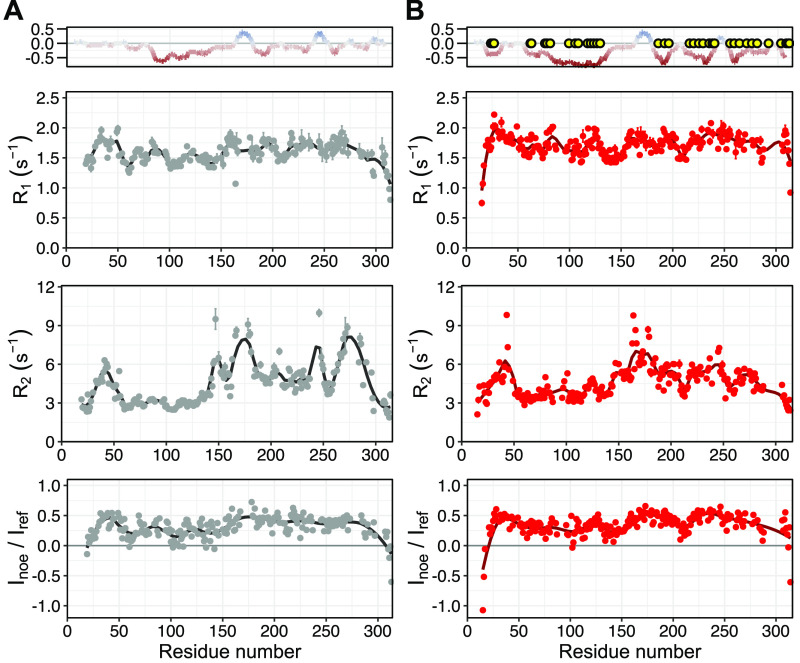
^15^N NMR relaxation data of OPN (A) before and (B) after phosphorylation, measured at 18.8 T. A charge plot of the protein sequence is shown at the top. Yellow circles indicate the identified phosphorylated residues. ^15^N *R*_1_, ^15^N *R*_2_, and ^15^N–{^1^H} NOE relaxation parameters, from top to bottom, respectively, of OPN measured at 293 K. Error bars indicate the fitting errors (^15^N *R*_1_ and ^15^N *R*_2_) and the error propagation of intensity ratios based on the noise level (hetNOE).

NMR observables such as chemical shifts or ^15^N relaxation rates are very informative for IDP structural dynamics.^51,52^ Possible changes in the structural dynamics of the protein were studied by a series of ^15^N relaxation experiments (Figure 3).

The ^15^N *R*_1_ patterns of both unphosphorylated and phosphorylated OPN show similar features, however with systematically larger *R*_1_ values for the modified protein. Interestingly, ^15^N *R*_2_ values of the residues in the second half of the protein (residues 200–314) decrease for the phosphorylated form, while fast NH vector motions are retained, as measured with ^15^N–{^1^H} NOE relaxation experiments. Overall, the region of residues 200–314 experiences an increase in backbone dynamics on the nanosecond time scale while faster picosecond time scale motions are nearly unaffected. In summary, the experimental data suggest enhanced dynamics in protein segments that comprise the majority of the phosphorylation sites. Further analysis, e.g., by applying the model free approach, was not pursued because connections between the measured phenomenological relaxation rates and the motions of a protein are far from trivial, especially for IDPs, where experimental rates are a mixture of polymer-like properties and non-uniform chain behaviors caused by secondary structure propensities, residue-dependent motions, and long-range correlated segments.^53^

### Phosphorylation Induces Structural Elongation of the Main Compact State in OPN

**Figure 4:**
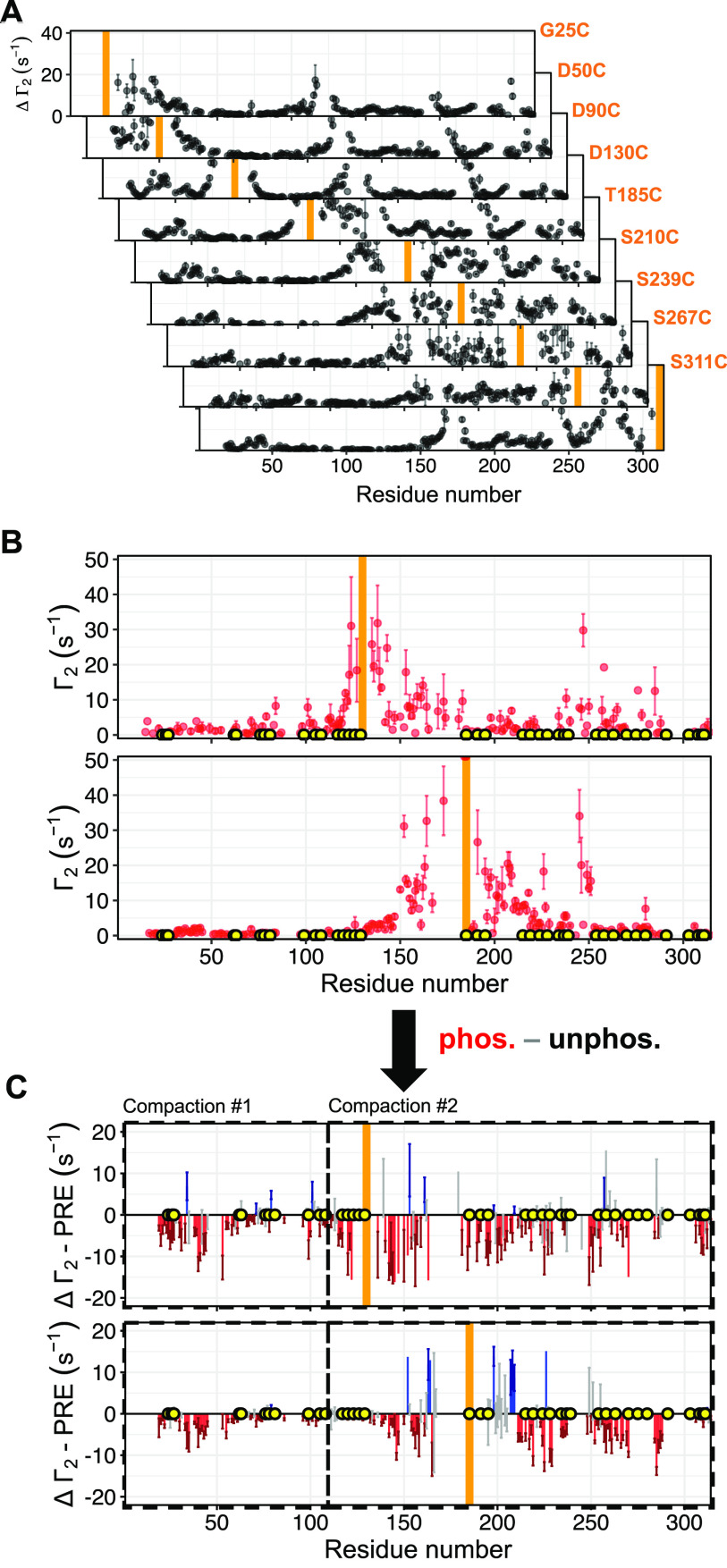
Effect of phosphorylation on long-range interactions measured by PRE experiments. (A) ^1^H^N^ Γ_2_ PRE profiles of different OPN cysteine mutants obtained from ^1^H^N^*T*_2_ NMR experiments. (B) ^1^H^N^ Γ^2^ PRE rates of the phosphorylated OPN mutants D130C (top) and T185C (bottom) determined from ^1^H^N^*T*_2_ NMR experiments. (C) Plot of the PRE rate difference of OPN and phosphorylated OPN mutants D130C (top) and T185C (bottom). Orange bars indicate the respective mutated cysteine residue with the attached spin-label.

Long-range structural contacts in unphosphorylated (Figure 4A) and hyperphosphorylated OPN (Figure 4B) were probed by measurements of PRE profiles for several cysteine mutants for unphosphorylated OPN, while the hyperphosphorylated state of OPN was probed using the two representative cysteine mutants D130C and T185C. In total, nine cysteine mutants were studied for the unphosphorylated OPN, which is necessary to overcome the intrinsic limitations of PRE measurements due to the *r*^–6^ averaging and to achieve a proper modeling of the long-range contacts, as shown elsewhere.^54^ Importantly, ^1^H *T*_2_ rates were measured instead of intensities, which adds a certain robustness to the experimental PRE data of IDPs. It is important to note that in the case of IDPs the measured *R*_2_ rates are the weighted population average, and therefore, conformations with greater *R*_2_ enhancements will be heavily weighted even if they are scarcely populated.^55^ A comparison of the PRE profiles obtained for cysteine mutants D130C and T185C clearly shows a striking reduction of long-range contacts within the central compact core region (residues 120–250), at the N-terminal region around residues 25–30, and within the whole C-terminus of the protein, while most of the negatively charged regions remain unaffected (Figure 4C). To conclude, our data suggest a significant structural elongation of OPN due to hyperphosphorylation, accompanied by an increase in local flexibility in the C-terminal region (residues 200–314) that is particularly rich in phosphorylation sites.

### Decrease in the Binding Affinity for Heparin Due to Phosphorylation while Retaining Striking Carbohydrate Specificity

**Figure 5:**
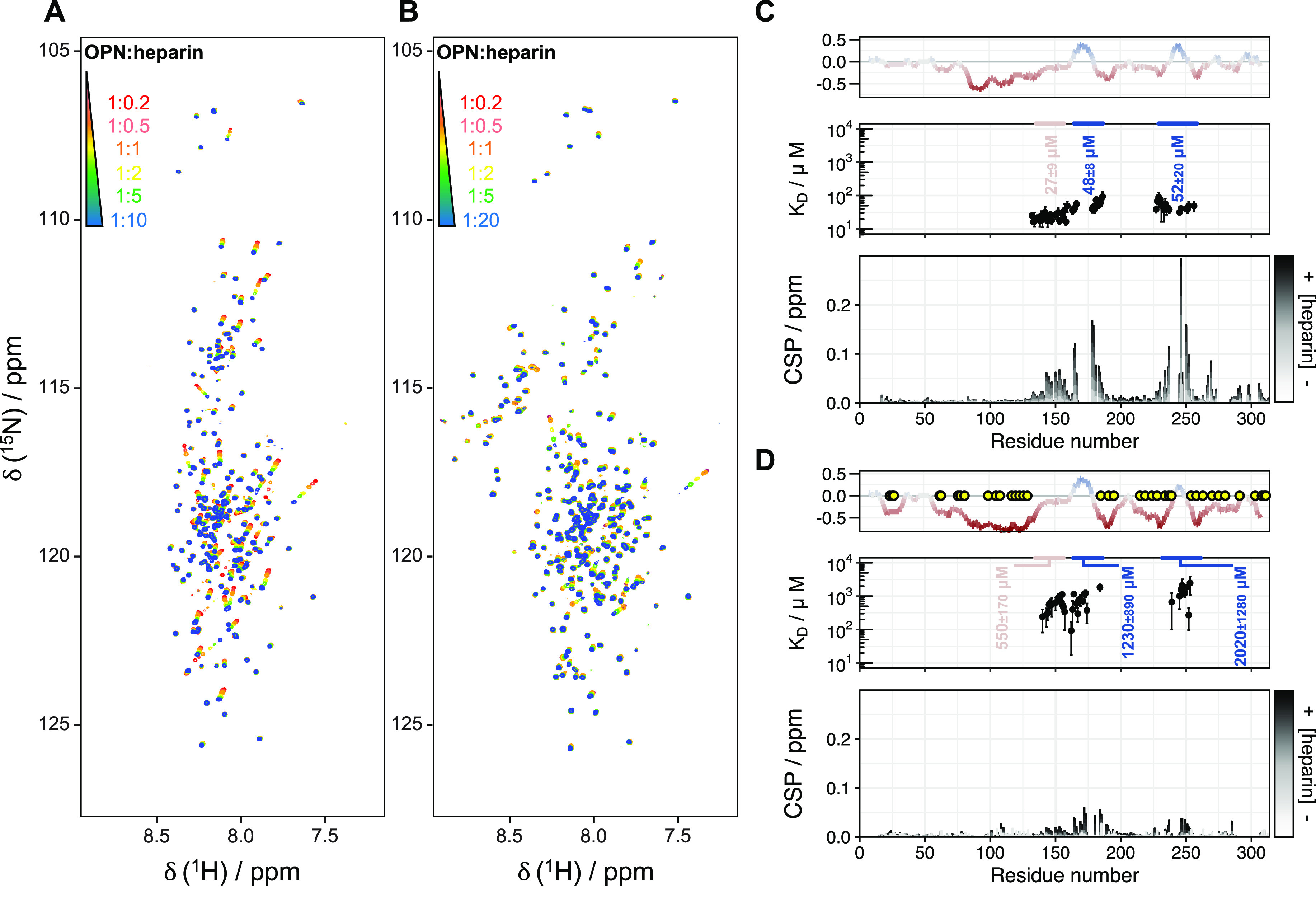
Binding of (phosphorylated) OPN to heparin, monitored by NMR titrations. ^1^H–^15^N HSQC NMR spectra in the presence of increasing amounts of heparin (red to blue) for the (A) unphosphorylated and (B) hyperphosphorylated forms. Chemical shift perturbations (bottom panel) and fitted *K*_D_ of binding regions (middle panel in blue) and the uncompacted region (middle panel in pink) plotted against the residue numbers of (C) OPN and (D) phosphorylated OPN. The corresponding charge plots are shown at the top. Yellow circles indicate the identified phosphorylated residues. The grayscale (from white to black) represents the increasing OPN:hep molar ratio from 1:0.2 to 1:10 (unphosphorylated) and 1:20 (phosphorylated).

The binding of OPN to heparin, a chemical mimic of the natural glycosaminoglycan heparan sulfate (HS) in the ECM, was investigated by a series of titration experiments employing ^1^H–^15^N HSQC NMR spectroscopy (Figure 5A,B). In the unmodified form of OPN, binding to heparin mainly induces chemical shift changes in the positively charged regions (residues 180–190 and 240–260), while modest chemical shift perturbations are found for residues located in the region of residues 140–160 (Figure 5C). A quantitative fit analysis reveals a binding affinity in the micromolar range (48 ± 8 and 52 ± 20 μM) for both positively charged regions (Figure 5C middle panel, blue; Figure S7) in accordance with ITC data from previous work on a protein homologue.^46^ The observed affinity is very similar to that of the quail homologue form.^46^ As previously reported, the chemical shifts observed in the region of residues 140–160 may arise from a local “unfolding-upon-binding” process that occurs when OPN binds to this polyanionic carbohydrate.^46^

This phenomenon, also known as “cryptic disorder”, is a widespread mechanism of folded proteins and IDPs in response to environmental changes (such as binding or protein modifications).^56,57^ Upon binding, the compensation of entropic loss (from a large conformational ensemble in the free form to a restricted set of conformations in the bound form) can be established in different mechanisms.^58^ Among them, IDPs may maximize the entropic gain by increasing the flexibility in regions distant from the binding sites, as it was reported for the mechanism of binding of OPN to heparin: a local rigidification in the heparin binding cleft (central core region) leads to a conformational entropy penalty that is reduced by a compensatory increase in the conformational flexibility of the negatively charged regions.^46^ Upon phosphorylation of OPN, the binding affinity is clearly reduced and consequently more heparin was needed to reach saturation (Figure 5B), presumably due to stronger electrostatic repulsions involving the numerous phosphorylation sites in the region of residues 240–260. Here, the entropic penalty of retaining a partially structured central region in phosphorylated OPN is accommodated by increasing the dynamics of charged regions. A similar mechanism was described for the mode of binding of Sic1 to Cdc4, where entropic compensatory events are also present.^59,60^ Quantitative analysis of the observed chemical shift changes for both positively charged regions reveals an approximately 20/40-fold decrease in affinity [1230 ± 890 and 2020 ± 1280 μM (Figure 5D)]. Besides that of heparin, the binding of OPN to hyaluronic acid (HA), another ubiquitous extracellular matrix glycosaminoglycan,^61,62^ was tested (Figure S8). HA is composed of *N*-acetylglucosamine and glucuronic acid and binds to the abundant extracellular receptor CD44 through a conserved HA binding domain (CD44_HABD_).^63,64^ OPN was also described as a binding partner of CD44 (with and without HA).^65^ Given the polyanionic nature of HA and heparin, a similar mode of binding to OPN can be anticipated. Surprisingly, however, NMR titration experiments show no binding between HA and OPN, in the unphosphorylated or in the phosphorylated form of the protein. Additionally, no binding to CD44_HABD_ is identified in the presence or absence of HA [HA forms a tight complex with CD44_HABD_ (Figure S9)]. This points to an interesting and unexpected (charge-independent) differential specificity of OPN toward the various glycosaminoglycans of the ECM and clearly questions the notion of OPN being a disordered protein lacking structural preformation. Moreover, it restricts the CD44 binding site of OPN to the disordered CD44 region, where the HS modification is present.

### Decompaction of OPN Due to Hyperphosphorylation Modulates the Binding Affinity for Integrins

**Figure 6:**
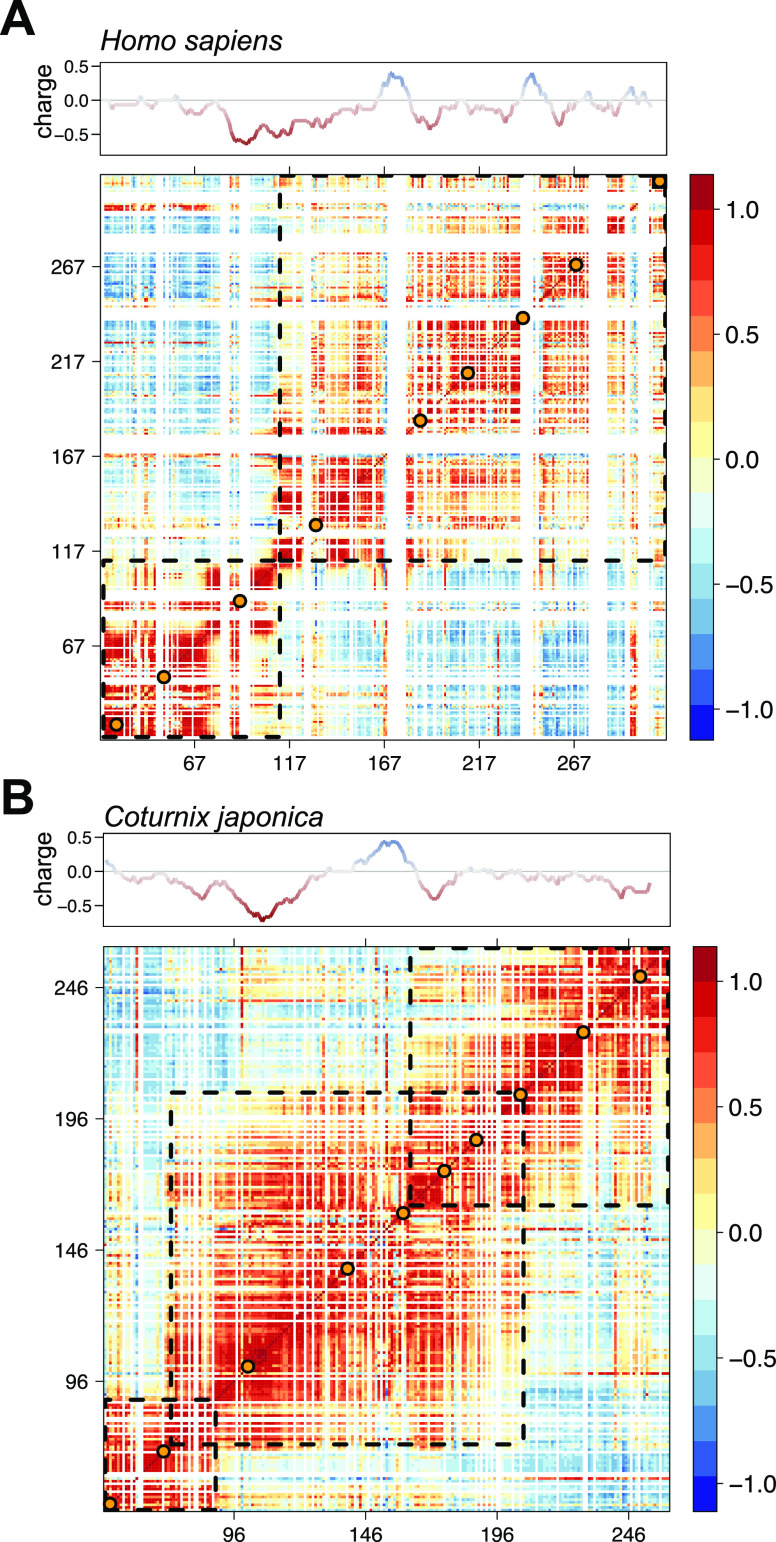
Pearson correlation maps of (A) *H. sapiens* OPN determined from nine PRE profiles and (B) *C. japonica* OPN determined from 10 PRE profiles. The maps show correlated (red to orange), uncorrelated (light yellow to light blue), and anticorrelated (light blue to dark blue) structural fluctuations. The dashed squares enclose regions of distinct structural compaction. The orange dots represent the spin-label sites. The data for the *C. japonica* OPN correlation matrix were previously published.^47^ Corresponding charge plots are shown at the top.

The seminal work of Tagliabbracci et al. on the characterization of Fam20C and its extracellular substrates reported the unexpected observation that Fam20C knockout MDA-MB-231 cells (i.e., no Fam20C-mediated phosphorylations) have superior adhesion properties.^21^ Moreover, Schytte et al. recently reported that the phosphorylation of an OPN construct, which covers the integrin binding motif, and full-length OPN, co-expressed with Fam20C, strongly hampers the interaction with α_v_β_3_ integrin.^66^ OPN is not only the most phosphorylated substrate of Fam20C but also a natural binder to integrin receptors.^48^ Thus, its phosphorylation may have a major impact on the mediation of cell–ECM adhesion properties through integrin binding. Recent studies of *Coturnix japonica* OPN showed that an expansion of the compact states due to rational mutations of the hydrophobic residues of the central core region leads to higher affinities for heparin^47^ and lower affinities for integrins. Both *C. japonica* OPN and *H. sapiens* OPN form compact central states exploiting electrostatic attractions between differently charged regions as well as backbone hydrophobic interactions.^67^ The existence of compact substates in OPN has been demonstrated.^46,47^ Correlated conformational fluctuations within the structure of both *H. sapiens* OPN and *C. japonica* OPN are visualized in a Pearson correlation map (Figure 6),^54,68^ derived from multiple PRE rates. *H. sapiens* OPN (Figure 6A) reveals two compacted regions (residues 14–115 and 116-314), whereas *C. japonica* OPN (Figure 6B) reveals three compacted regions (residues 46–90, 80–200, and 160–247). Interestingly, the residue segments of *H. sapiens* OPN, where the phosphorylation sites are located, show significant correlations. Therefore, we conclude that (hyper)phosphorylation of OPN releases long-range correlations (by weakening stabilizing/attractive electrostatic interactions) and leads to the observed decompaction. Thereby, it abolishes energetically favorable interactions between OPN sites that are distant from the canonical RGD motif and integrin receptors.^48,66^ However, the central part that contains the (integrin binding) RGD motif (residues 159–161) retains its local rigidity (^15^N *R*_2_ rates) and maintains a preformed template for receptor recognition.

## Concluding Remarks

In conclusion, we investigated the effect of phosphorylation of OPN by Fam20C. To this end, an optimized protocol for *in vitro* phosphorylation has been developed using Fam20C expressed in mammalian HEK293T cells. Furthermore, almost complete assignment of phosphorylated S-x-E/pS motifs has been achieved by a combination of MS and NMR spectroscopy. NMR studies of the hyperphosphorylated OPN reveal an increase in flexibility in regions, which comprise the Fam20C phosphorylation sites, and weakened long-range interactions. The role of electrostatics and side chain–backbone interactions has emerged recently as a potential mechanism for modulating the formation of rigid segments and overall compaction.^47,69^ Moreover, weak side chain–backbone interactions involving proline residues are important for stabilizing OPN’s central compact state. Most importantly, the observed decompaction is also in accordance with the reported biological behavior of OPN (a decreased binding affinity for integrins) and illustrates the importance of compact states for molecular recognition events in which IDPs are involved. However, it is challenging to address the degree of decompaction in a quantitative manner because it is extremely difficult to know the extent to which those conformations are populated. At the same time, the existence of functional minor populations (or excited stats) in IDPs may play a key role in binding events. Low-resolution techniques such as SAXS may not fully grasp the subtleties of IDP ensembles. On the contrary, PRE data accentuate the minor populations that seem to be relevant for understanding OPN function. Furthermore, the unexpected proteoglycan binding preference (heparan sulfate vs hyaluronic acid) of OPN suggests an interaction specificity of IDPs and questions the notion of IDPs being fully disordered and exhibiting random-coil type behavior. To conclude, post-translational modifications, in our case phosphorylation, are effective mechanisms for modifying conformational ensembles of IDPs and populating suitable substates for molecular recognition events. Structural disorder is clearly not adequate for grasping the subtlety of these processes, and more sophisticated concepts have to be involved to fully appreciate how IDPs can respond to changing molecular environments and how they can engage in permanently varying protein interaction networks.

## Supporting Information Available

Materials and Methods, including protein/kinase production, protein phosphorylation, labeling, NMR spectroscopy, and MS experimental details; affinity column chromatogram of OPN; anion exchange chromatogram of (phosphorylated) OPN; sodium dodecyl sulfate–polyacrylamide gel electrophoresis and Western plot of Fam20C; phosphopeptide identification by MS; phosphoresidue identification by MS and NMR; secondary shift propensity of phosphorylated OPN; selectred NMR titration curves of OPN:heparin; series of ^1^H–^15^N HSQC NMR spectra of OPN:HA; ^1^H NMR spectra of OPN[+CD44_HABD_(+HA)]; and tables of backbone chemical shifts of phosphorylated OPN (BMRB ID: 50447)(PDF)
Tables of MS results for unphosphorylated OPN (XLSX)
Tables ofMS results for phosphorylated OPN (XLSX)

## Accession Codes

Human OPN, P10451 (Uniprot); quail OPN, Q9I832 (Uniprot); human Fam20C, Q8IXL6 (Uniprot).
