# Supplementary material for: Hyperphosphorylation of Human Osteopontin and Its Impact on Structural Dynamics and Molecular Recognition
Source: Biochemistry. 2021 Apr 20;60(17):1347–55. doi: 10.1021/acs.biochem.1c00050 (PMC8154273; doi:10.1021/acs.biochem.1c00050)
Supplement: Supplementary file 1 — bi1c00050_si_001.pdf [file bi1c00050_si_001.pdf]

# Supplementary information

# Hyperphosphorylation of Human Osteopontin and its Impact on Structural Dynamics and Molecular Recognition

Borja Mateos[a,1], Julian Holzinger[a,1], Clara Conrad-Billroth[a], Gerald Platzer[a], Szymon Żerko[b], Marco Sealey-Cardona[c], Dorothea Anrather[d], Wiktor Koźmiński[b] and Robert Konrat[a,*]

[a] Department of Structural and Computational Biology, University of Vienna, Max Perutz Labs, Vienna Biocenter Campus 5, 1030 Vienna, Austria.

[b] Faculty of Chemistry, Biological and Chemical Research Centre, University of Warsaw, 02093, Warsaw, Poland

[c] CALYXHA Biotechnologies GmbH, Karl-Farkas-Gasse 22, 1030 Vienna, Austria.

[d] Mass Spectrometry Facility, Max Perutz Labs, Vienna Biocenter Campus 5, Dr. Bohr Gasse 3, Vienna, 1030, Austria.

[1]B.M. and J.H. contributed equally to this work

*To whom correspondence may be addressed. Email: robert.konrat@univie.ac.at

**This SI contains:**

Materials and Methods………………………………………………………… 3

1. *Homo sapiens* Osteopontin production……………………….. 3
2. Human Fam20C kinase production………………….....……… 4
3. *In vitro* Osteopontin phosphorylation by Fam20C…..……….. 5
4. MTSL labelling……………………………………………..…..... 6
5. NMR spectroscopy……………………………………...……….. 6
6. Mass spectrometry……………………………………...……….. 7

Figure S1…………………………………………………………………... 9
Figure S2…………………………………………………...……………... 10
Figure S3…………………………………………………...……………... 11
Figure S4…………………………………………………...……………... 12
Figure S5…………………………………………………...……………... 13
Figure S6…………………………………………………...……………... 14
Figure S7…………………………………………………...……………... 15
Figure S8…………………………………………………...……………... 16

Table S1…………………………………………………...……………... 17
Table S2…………………………………………………...……………... 21
Table S3…………………………………………………...……………... 22

SI References……………………………………………………………….. 23

## 1. *Homo sapiens* Osteopontin production

```
MRIAVICFCL LGITCAIPVK QADSGSSEEK QLYNKYPDAV ATWLNPDPSQ  50
KQNLLAPQNA VSSEETNDFK QETLPSKSNE SHDHMDDMDD EDDDDHVDSQ  100
DSIDSNDSDD VDDTDDSHQS DESHHSDESD ELVTDFPTDL PATEVFTPVV  150
PTVDTYDGRG DSVVYGLRSK SKKFRRPDIQ YPDATDEDIT SHMESEELNG  200
AYKAIPVAQD LNAPSDWDSR GKDSYETSQL DDQSAETHSH KQSRLYKRKA  250
NDESNEHSDV IDSQELSKVS REFHSHEFHS HEDMLVVDPK SKEEDKHLKF  300
RISHELDSAS SEVN                                         314
```

**Signal peptide (SP)**

**Integrin RGD binding motif**

In pETM-11: $His_6$-TEV-$OPN_{17\text{-}314}$

```
MKHHHHHHPMSDYDIPTTENLYFQ.GAMGIPVKQADSGSSEEKQLYNKYPDAVATWLNPDPSQK
QNLLAPQNAVSSEETNDFKQETLPSKSNESHDHMDDMDDEDDDDHVDSQDSIDSNDSDDVDDTD
DSHQSDESHHSDESDELVTDFPTDLPATEVFTPVVPTVDTYDGRGDSVVYGLRSKSKKFRRPDI
QYPDATDEDITSHMESEELNGAYKAIPVAQDLNAPSDWDSRGKDSYETSQLDDQSAETHSHKQS
RLYKRKANDESNEHSDVIDSQELSKVSREFHSHEFHSHEDMLVVDPKSKEEDKHLKFRISHELD
SASSEVN
```

*Transformation.* Approximately ~100 ng of the hOPN (UniProt: P10451) encoding DNA vector (residues 17-314, signal peptide removed) was mixed with an aliquot of *Escherichia coli* BL21(DE3)Rosetta$^{TM}$ pLysS phage resistant strain. It was kept 15 min on ice, afterwards, for 1 min at 42ºC and 2 min on ice again. Finally, 300 µL of LB media were added and the mixture was incubated at 37ºC for 45-60 min under agitation. 50 µL were plated on a kanamycin+chloramphenicol agar plate and incubated overnight (o/n) at 37ºC.

*Preculture.* A colony from the plate was picked and inoculated in 100 mL of LB medium with kanamycin+chloramphenicol at 1:1000 dilution (1:2000 of each antibiotic) (LB-Kan+Cam). It was incubated o/n at 37ºC under agitation (140 rounds per minute (rpm)).

*Expression.* 10mL of the preculture was added to 3 L of LB-Kan+Cam. The expression flasks were incubated at 37ºC under agitation (140rpm) until the $OD_{600}$ had reached 0.6-0.8. If $^{15}N/^{13}C$ labelling was needed, the cells were pelleted at 5000 rpm for 15 min at 25ºC, the supernatant was discarded and the pellet resuspended in 1 L of $^{15}N/^{13}C$-enriched M9 media, as proposed elsewhere[1]. The cell expression was induced with IPTG at a final concentration of 0.4 mM from a 0.4 M stock solution (1 mL in 1 L of $^{15}N/^{12}C$ LB or $^{15}N/^{13}C$ M9 media). The expression culture was left at 28ºC under 140 rpm agitation o/n.

*Harvesting.* The cells were harvested by centrifugation at 5000 rpm for 15 min at 5ºC. The supernatant was discarded and the pellet was resuspended with ice-cold PBS buffer (40 mL per litre of culture) supplemented with one aliquot of Halt™ Protease Inhibitor cocktail (*ThermoFisher*). 2 mM of β-Mercaptoethanol (β-Mer) was added in case of working with a cysteine mutant (for PRE measurements).

*Purification.* The harvested cells were sonicated 3x3 min (50% on/off). Afterwards, the suspension was centrifuged at 18000 rpm for 45 min at 5ºC. The pellet was discarded, and the supernatant was cooked at 75ºC for 10 min and subsequently centrifuged at 18000 rpm for 45 min at 5ºC. The pellet was discarded again, and the supernatant loaded with 1 mL/min in a HisTrapFF crude column (*GE lifesciences*) for an affinity column purification step. After loading, the column was washed with PBS buffer (pH = 8) with 5 mL/min until the baseline is reached again. The protein was eluted with an imidazole gradient from 0 to 100% (500mM imidazole) with 1 mL/min. during 30 min. The protein elutes with a decent purity grade at this stage, as tested by SDS-PAGE (**Figure S1**).

All the fractions containing the protein after the affinity column were collected and concentrated to 0.5-1 mL using a 10 kDa cut-off Amicon® Ultra centrifugal unit (*Merck*). The buffer was exchanged to the TEV cleavage buffer (1xPBS, 1 mM DTT and 1 mM Ethylenediaminetetraacetic acid (EDTA)). The protein was incubated with TEV protease (1 mg per 50 mg of protein) o/n at 4ºC. 1 mM of DTT was added in case of working with a cysteine mutant (i.e. for PRE measurements). Further purification was achieved by loading the sample onto a 5 mL HiTrapQ HP anion exchange column (*GE Healthcare*) with 1-2 mL/min. The conductivity should not exceed 18 mS/cm during the sample load in order to prevent a premature protein elution. The protein was eluted with a NaCl gradient from 0% to 50% (500 mM NaCl) during 30 min with 1 mL/min. The protein elutes at a conductivity of 37-39 mS/cm (**Figure S2**).

*Final preparation.* The fractions containing the protein were concentrated and buffer exchanged using a 10kDa cut-off Amicon® Ultra centrifugal unit (*Merck*). The protein concentration was calculated by spectrophotometry using a NanoDrop™ (*ThermoFisher*). The absorbance ratio 260/280 should be lower than 0.7 to be sure of not having DNA contaminants. For each 1 L of starting LB culture approx. 1 mL of a 0.2 mM purified sample could be obtained.

*2.* Human Fam20C kinase production

The HEK293T cells lines that constitutively express Fam20C or a catalytically inactive mutant (Fam20C D478A) were kindly provided by Vincent S. Tagliabracci (UT Southwestern Medical Center, Texas, United States of America). The protocol performed for the production of this kinase is a modified version of his original paper.[2] The mutant D478A was used as a negative control in the phosphorylation reaction (see supplementary raw data of MS analysis).

The stably expressing HEK293T cell lines were grown for 84-96h to reach confluency in Dulbecco's Modified Eagle Medium (DMEM) supplemented with 10% of Fetal Bovine Serum (FBS) and 1% of Penicillin-Streptomycin (Pen-Strep) in 4xT175 flasks (*Thermo Fisher*). Since this protein has a secretion signal peptide, the medium contains high amounts of kinase. The conditioned medium was removed, centrifuged at 5000 rpm for 5 min at 4ºC, passed through a 0.45 µm filter and one aliquot of Halt™ Protease Inhibitor cocktail (*ThermoFisher*) was added while keeping the temperature. Afterwards, Anti-Flag M2-agarose (*Sigma-Aldrich*) was added and incubated o/n at 4ºC. The solution was loaded onto a 20 mL column and washed with phosphorylation buffer (50 mM Tris, 50 mM NaCl, pH = 7.2). The resin was incubated with 1 mL of elution buffer (phosphorylation buffer + 100 µg/mL of FLAG peptide (*Sigma-Aldrich*)) for 4 h under agitation before elution. The elution step was repeated three times. The eluents were kept in 5% glycerol and 2 mM of β-Mer, snap-frozen with $N_{2(l)}$ and stored at -80°C until use. The typical yield of FLAG-tagged Fam20C using this procedure was approximately 1-1.5 mg protein per liter of medium. Sample purity was checked by SDS-PAGE and Western blot (**Figure S3**).

*3. In vitro* Osteopontin phosphorylation by Fam20C

A purified aliquot of hOPN was exchanged to phosphorylation buffer (50 mM Tris, 50 mM NaCl, pH = 7.2).

The phosphorylation reaction was run in a reaction volume of 2 mL, including 50 µM hOPN, 300 µL of eluted Fam20C and 2 mM β-Mer. ATP (5 µmol) and $MnCl_2$ (18 µmol) were added at the start of the reaction and afterwards approx. every 4 h except o/n, in total resulting in a reaction time of 48h, 30 µmol ATP and 90 µmol $MnCl_2$. It is important to note that $Mn^{2+}$ coordinates with the inorganic phosphate generated in the course of the reaction and the resulting OPNp. The reaction was stopped by the addition of EDTA (1 mM) until no precipitation was observable anymore. The phosphorylated OPN sample was

exchanged to NMR measurement buffer (NaP 50 mM, NaCl 50 mM, $NaN_3$ 1 mM, EDTA 1 mM) using a 10 kDa cut-off Amicon® Ultra centrifugal unit (*Merck*). The degree of phosphorylation was checked by $^{1}H$-$^{15}N$ HSQC NMR spectroscopy.

In case the sample was not phosphorylated completely, it was exchanged back to phosphorylation buffer and the phosphorylation was performed once more (however only with approx. 100 μL Fam20C per mL of reaction volume).

After complete phosphorylation, the sample was exchanged to TEV cleavage buffer using a 10 kDa cut-off Amicon® Ultra centrifugal unit (*Merck*) or a PD-10 desalting column (*GE lifesciences*) and reloaded in a 5 mL HiTrapQ HP anion exchange column (*GE Healthcare*). The increased number of charges increments the affinity of the protein to the column and it elutes at conductivities around 45 mS/cm (**Figure S2**).

*4.* MTSL labelling

For the MTSL-tagging, cysteine mutants of (phosphorylated) OPN were incubated with an excess of DTT (10 mM) for 15 min at room temperature. A PD-10 Desalting-Column was equilibrated with 100 mM sodium phosphate, 1 mM EDTA, and pH = 8.0 buffer, the protein loaded to a total volume of 2.5 mL and eluted with 3.4 mL of buffer. The free thiol concentration was measured using a 300 mM DTNB (5,50-dithiobis-(2-nitrobenzoic acid)) solution at 412 nm. A threefold excess of *S*-(1-oxyl-2,2,5,5-tetramethyl-2,5-dihydro-1H-pyrrol-3-yl) methyl methane- sulfonothioate (MTSL) was added and the sample was incubated for 3 h at 37 ºC under agitation. The free thiol concentration was measured again to confirm complete tagging of the protein. Protein purity was confirmed via SDS-PAGE electrophoresis. MTSL is a nitroxide spin radical providing the paramagnetic form. The diamagnetic form is achieved by reducing the nitroxide group with ascorbic acid.

*5.* NMR spectroscopy

*Assignment*. The NMR experiments used for sequence specific assignment of $H^N$, N, C´, $C^\alpha$, $C^\beta$ resonances of the phosphorylated OPN were recorded at 293K. BMRB deposited shifts (ID: 50447) can be found in **Table S1**. Assignment experiments were acquired with a Bruker Avance III HD+ 800MHz spectrometer equipped with a room-temperature TXI-probehead. The backbone assignments were obtained using BEST-TROSY type versions of HNCACB, HN(CO)CACB, HN(CA)NNH, HN(COCA)NNH, HNCO, and HN(CA)CO

experiments.[3] The assignment of the phosphorylated hOPN resonances was conducted employing the CCPNmr software package.[4] The secondary structure propensity (SSP) score was estimated as proposed by Marsh et al.[5] and using random-coil shifts extracted from POTENCI.[6] Acquisition parameters are detailed in the **Table S2**.

*Paramagnetic Relaxation Enhancement*. The $^1H^N$-$R_2$ rates for (phosphorylated) OPN were acquired in a Bruker Avance III HD+ 800MHz spectrometer at 293K. The pulse sequence used was a pseudo-3D $^1H$-$T_2$ HSQC. Six relaxation delays (pseudo-dimensions) were acquired to fit an exponential decay function (0.001, 0.005, 0.01, 0.02, 0.05 and 0.1 s) and extract the $^1H$-$R_2$ ($R_2$=1/$T_2$). Acquisition parameters are detailed in the **Table S3**.

The paramagnetic effects were quantified according to the following formula:

$$\Delta(^1H^N)\Gamma_2 = (^1H^N)R_{2,para} - (^1H^N)R_{2,dia}$$

The correlation maps were built with RStudio. Pearson correlation (ρ) between two variables (PRE rates x and y for a residue i) was calculated as:

$$\rho_{X,Y} = \frac{\frac{1}{n}\sum_{i=1}^{n}(x_i - \bar{x})(y_i - \bar{y})}{\sigma_X\sigma_Y} = \frac{cov_{x,y}}{\sigma_X\sigma_Y}$$

Where $\sigma_X$ and $\sigma_Y$ are the standard deviations of the variables and 'cov' means covariance.

*$^{15}N$-relaxation*. NMR experiments were recorded on an 18.8 T Bruker Avance III HD+ spectrometer operating at 800 MHz. The $^{15}N$-$T_1$ relaxation delays were 0.01, 0.02, 0.04, 0.08, 0.16, 0.32, 0.64 and 1.28 s. The $^{15}N$-$T_2$ relaxation delays were 0, 0.017, 0.034, 0.068, 0.136, 0.271, 0.407 and 0.543 s. $^{15}N$-{$^1H$} heteronuclear NOE was measured with an interscan delay of 10 s to avoid systematic errors in the measurement of steady-state NOE as a consequence of incomplete equilibration of the magnetization in the reference spectrum[7]. Acquisition parameters are detailed in **Table S3**.

*Heparin titration.* The chemical shift perturbations from OPN:heparin titration NMR experiments were analyzed and fitted with an equation for species in fast chemical exchange, from which the affinities $K_D$ were determined:

$$CSP = \frac{\delta_{max}(([P]_{tot} + [L]_{tot} + K_D) - \sqrt{([P]_{tot} + [L]_{tot} + K_D)^2 - 4[P]_{tot}[L]_{tot}})}{2[P]_{tot}}$$

Figure S7 shows the fit analysis performed for a selected set of residues from the regions with the largest chemical shift perturbations.

*6.* Mass spectrometry

All the mass spectrometry experiments were carried out at the MS facility of the Max Perutz Labs. Intact mass experiments were used to test the purity of the samples and to determine the mass of the purified proteins. The LC-MSMS setup was used to identify the phosphorylation sites of OPN after incubation with Fam20C.

*Intact mass.* 60 to 80 ng of the purified protein were loaded onto an AERIS C4 widepore column (3.6 µm particle size, dimensions 2.1 × 150 mm; Phenomenex) using a Dionex Ultimate 3000 HPLC system (Thermo Fisher Scientific) with a working temperature of 50 °C, running a step gradient 9-36-63% acetonitrile (ACN) in 0.1% formic acid (FA) at a flow rate of 300 µL/min. The HPLC was coupled to a Synapt G2-Si via a ZSpray ESI source (Waters). Data were recorded with MassLynx V 4.1 (Waters) and analyzed using the MaxEnt 1 process to reconstruct the uncharged average protein mass.

*Phosphorylation mapping by LC-MS/MS*. Phosphorylated OPN was digested in solution with trypsin over night at 37 °C at a 1:30 enzyme-protein ratio. Digestion was stopped by adding trifluoroacetic acid to a final concentration of approximately 1% and the peptides were desalted using custom-made C18 stagetips[8]. For chromatographic separation on an UltiMate 3000 HPLC system (Dionex, Thermo Fisher Scientific) peptides were loaded onto a trapping column (PepMap C18, 5 µm particle size, 300 µm i.d. × 5 mm; Thermo Fisher Scientific) equilibrated with 0.1% TFA and separated on an analytical column (PepMap C18, 3 µm, 75 µm i.d. × 150 mm; Thermo Fisher Scientific), applying a 120 min linear gradient from 1.6% up to 28% ACN with 0.1% FA. Eluting peptides were analysed on a Q Exactive HF Orbitrap mass spectrometer equipped with a Proxeon nanospray source (Thermo Fisher Scientific), operated in a data-dependent mode. Survey scans were obtained in a scan range of 375–1500 m/z, at a resolution of 120000 at 200 m/z and an AGC target value of 3E6. The 8 most intense ions were selected with an isolation width of 1.6 Da, fragmented in the HCD cell at 27% collision energy and the spectra recorded at a target value of 1E5 and a resolution of 30000. Peptides with a charge of +1 were excluded from fragmentation, the peptide match and exclude isotope features were enabled and selected precursors were dynamically excluded from repeated sampling for 30s.
Raw data were processed using the MaxQuant software package (version 1.6.0.16, http://www.maxquant.org/)[9] and searched against the sequences of the hOPN construct, the Uniprot E.coli reference database (www.uniprot.org) and the MaxQuant common contaminant database. The search was performed

with trypsin specificity and a maximum of two missed cleavages. Oxidation of methionine, protein N-terminal acetylation and phosphorylation of serine, threonine and tyrosine were set as variable modifications - all other parameters were set to default. Results were filtered at a false discovery rate of 5% at the peptide and protein level.

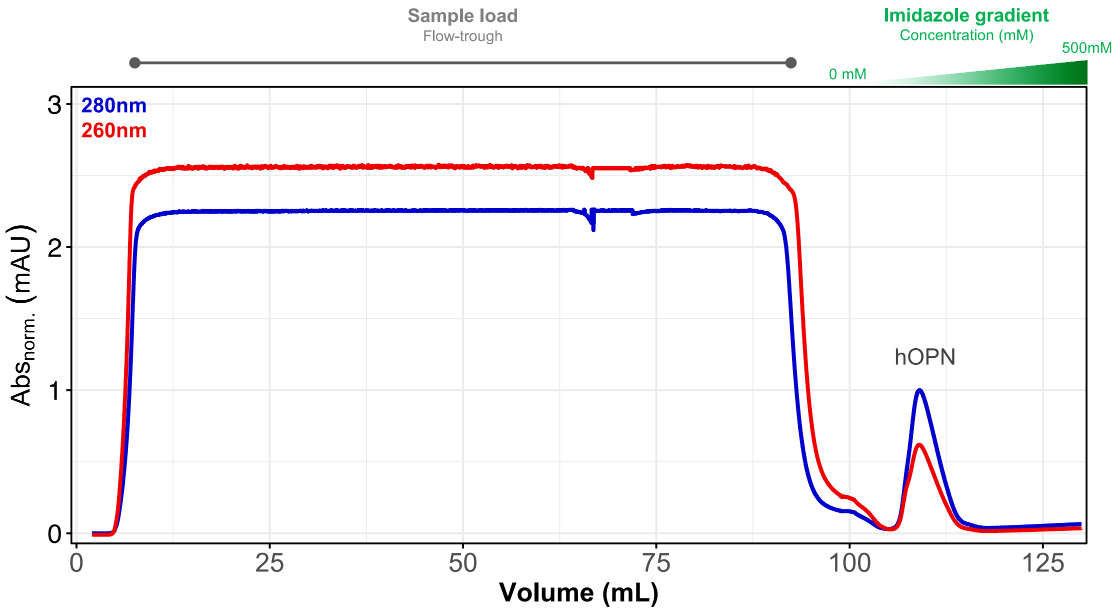


**Figure S1.** Typical affinity column (HisTrap) chromatogram of OPN. The blue and red lines indicate the absorbance at 280nm and 260nm wavelength, respectively. The absorbance values were normalized by the highest 280nm value of the protein peak.

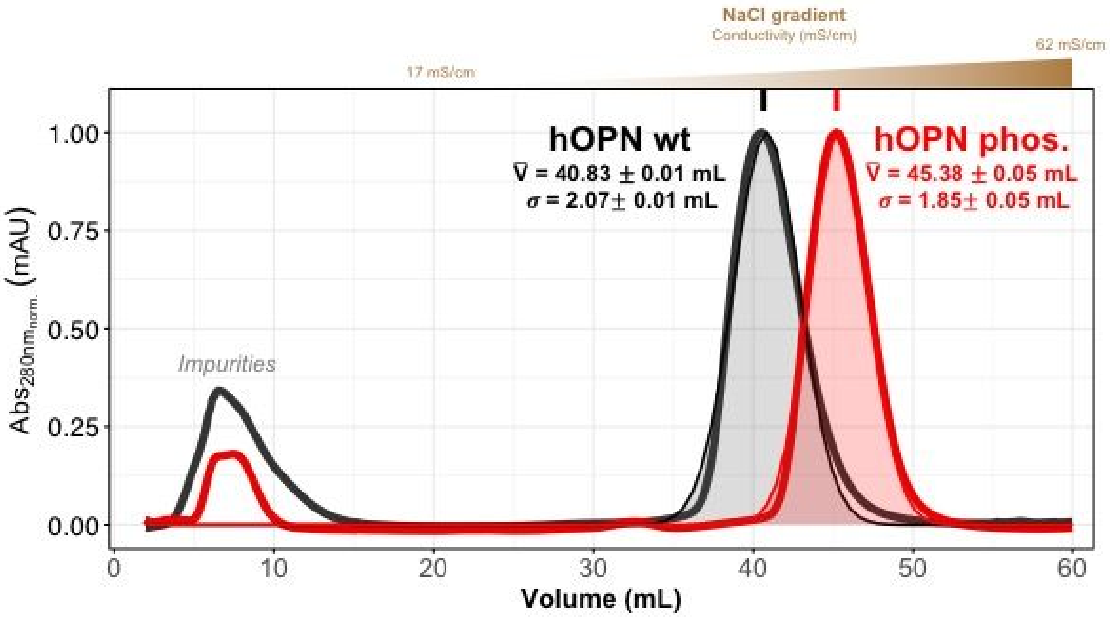


**Figure S2.** Anion exchange chromatogram of OPN. The lines indicate the absorbance at 280nm wavelength of the unphosphorylated (black) and phosphorylated (red) protein, respectively. The absorbance values were normalized by the highest value of the protein peak. Fitted Gaussian distributions are shown with shadowed areas. Unphosphorylated and phosphorylated OPN elute at approximately conductivities of 37 and 45mS/cm, respectively. Some impurities such as TEV protease and other phosphorylation crude reaction components elute during the sample load step.

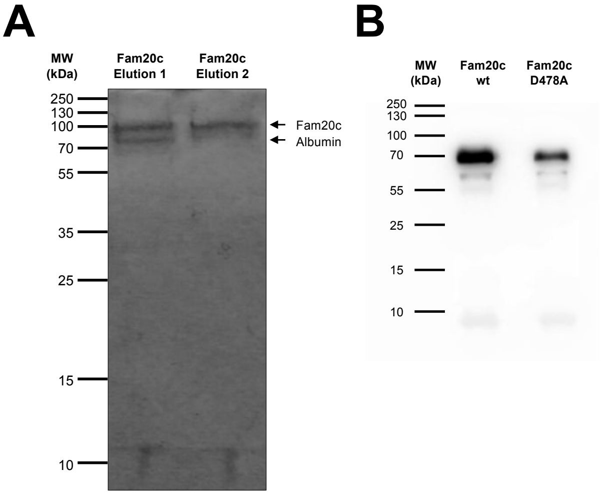


**Figure S3.** (A) SDS-PAGE of the FLAG-tagged Fam20C eluents. (B) Western-blot (anti-FLAG primary antibody) of the Fam20C wt and the catalytically inactive mutant D478A.

MRIAVICFCL LGITCAIPVK QADSGSSEEK QLYNKYPDAV ATWLNPDPSQ KQNLLAPQNA VSSEETNDFK QETLPSKSNE

coverage MRIAVICFCL LGITCAIPVK QADSGSSEEK QLYNKYPDAV ATWLNPDPSQ KQNLLAPQNA VSSEETNDFK QETLPSKSNE
P-peptides MRIAVICFCL LGITCAIPVK QADSGSSEEK QLYNKYPDAV ATWLNPDPSQ KQNLLAPQNA VSSEETNDFK QETLPSKSNE

SHDHMDDMDD EDDDDHVDSQ DSIDSNDSDD VDDTDDSHQS DESHHSDESD ELVTDFPTDL PATEVFTPVV PTVDTYDGRG

coverage SHDHMDDMDD EDDDDHVDSQ DSIDSNDSDD VDDTDDSHQS DESHHSDESD ELVTDFPTDL PATEVFTPVV PTVDTYDGRG
P-peptides SHDHMDDMDD EDDDDHVDSQ DSIDSNDSDD VDDTDDSHQS DESHHSDESD ELVTDFPTDL PATEVFTPVV PTVDTYDGRG

DSVVYGLRSK SKKFRRPDIQ YPDATDEDIT SHMESEELNG AYKAIPVAQD LNAPSDWDSR GKDSYETSQL DDQSAETHSH

coverage DSVVYGLRSK SKKFRRPDIQ YPDATDEDIT SHMESEELNG AYKAIPVAQD LNAPSDWDSR GKDSYETSQL DDQSAETHSH
P-peptides DSVVYGLRSK SKKFRRPDIQ YPDATDEDIT SHMESEELNG AYKAIPVAQD LNAPSDWDSR GKDSYETSQL DDQSAETHSH

KQSRLYKRKA NDESNEHSDV IDSQELSKVS REFHSHEFHS HEDMLVVDPK SKEEDKHLKF RISHELDSAS SEVN

coverage KQSRLYKRKA NDESNEHSDV IDSQELSKVS REFHSHEFHS HEDMLVVDPK SKEEDKHLKF RISHELDSAS SEVN
P-peptides KQSRLYKRKA NDESNEHSDV IDSQELSKVS REFHSHEFHS HEDMLVVDPK SKEEDKHLKF RISHELDSAS SEVN

**Figure S4.** Schematic representation of identified phosphopeptides (grey boxes) by MS.

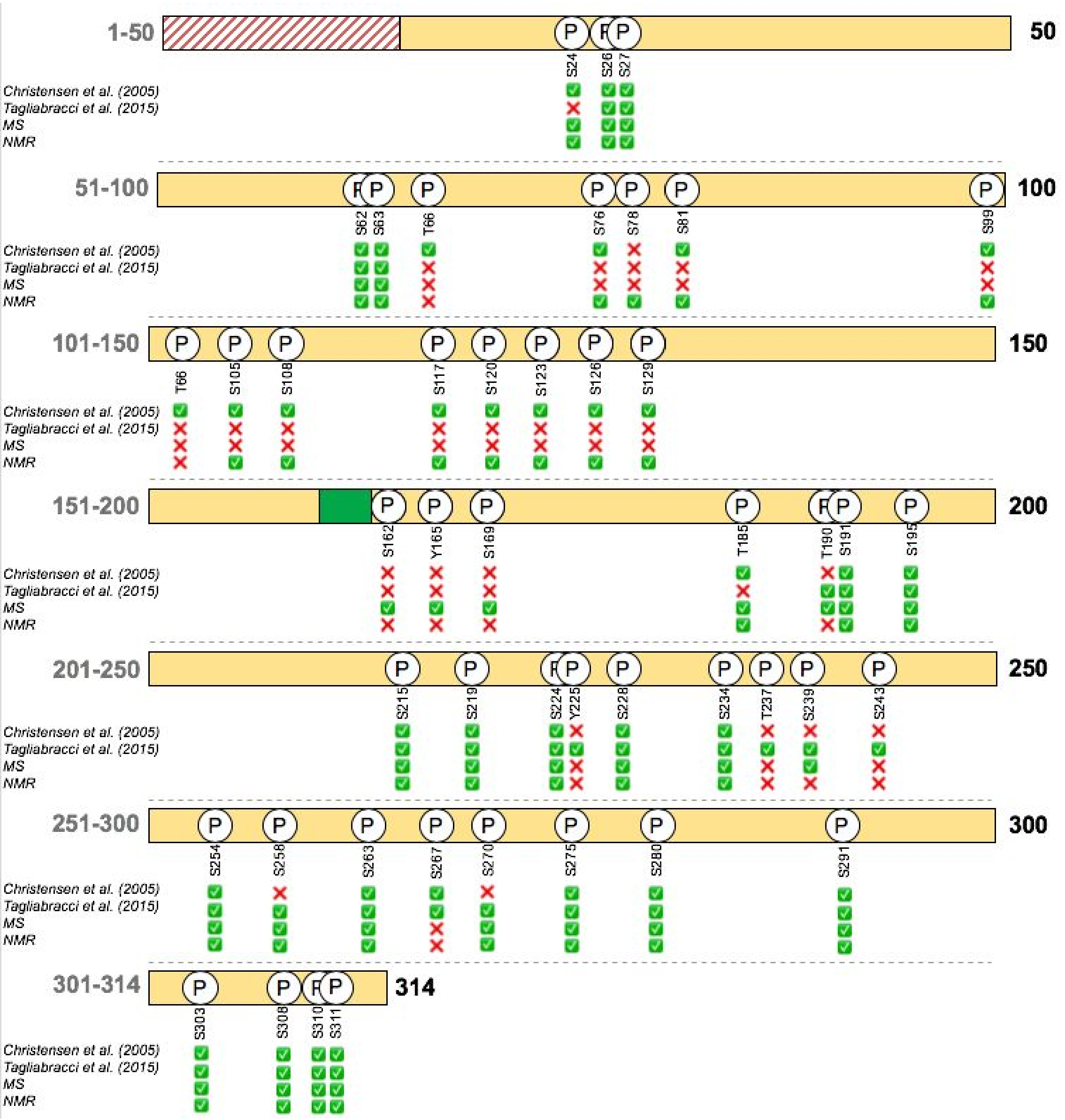


**Figure S5.** Schematic representation of phosphoresidues identified by MS and NMR spectroscopy compared with previously reported ones[10,11].

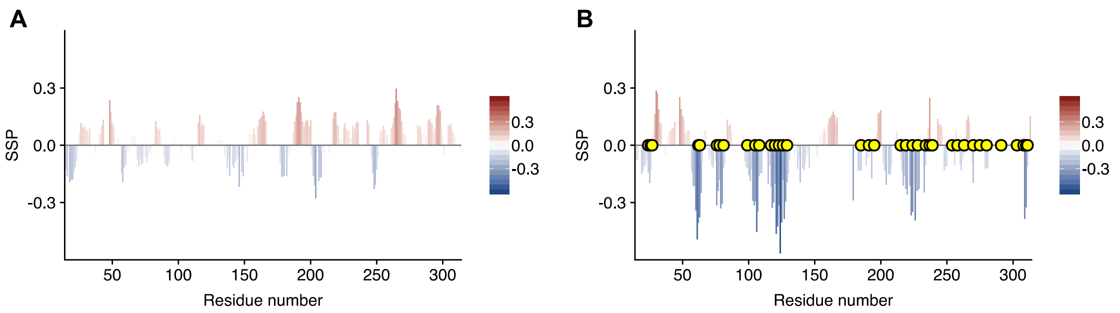


**Figure S6.** Secondary shifts propensity (SSP) values of OPN before (A) and after (B) phosphorylation.[5] Positive values (red) are associated with α-helix and negative values (blue) indicate a propensity to generate β-strand conformations. Yellow circles indicate the identified phosphorylation sites.

NOTE: The $C^{\alpha}$ and $C^{\beta}$ chemical shifts are excellent reporters of residual secondary structure in IDPs.[5,6,12] Assigned backbone and sidechain chemical shifts were used to investigate the effect of phosphorylation on the secondary structure of the protein. The hyperphosphorylated form displays lower secondary structure propensity (SSP) values in comparison to the unphosphorylated form of the protein.[13] However, recent publications with random-coil shifts of modified aminoacids[14,15] reported on how the $C^{\beta}$ chemical shifts of serines and threonines experience a downfield shift of around 3–4 ppm due to the proximity of this atom to the phosphate group. These downfield shifts were generally also observed in the context of the full-length OPN upon phosphorylation and it explains the biasing of SSP towards negative values (extended conformations). It is important to note that the region around the RGD integrin-binding motif ($^{159}$RGD$^{161}$) does not experience any change regarding the residual structure, largely due to the lack of phosphorylation sites in the vicinity. Thus, the apparent extension of OPN conformations in SSPs (i.e. adopting more beta-strand element populations) is triggered by the drastic $C^{\beta}$ downfield shift.

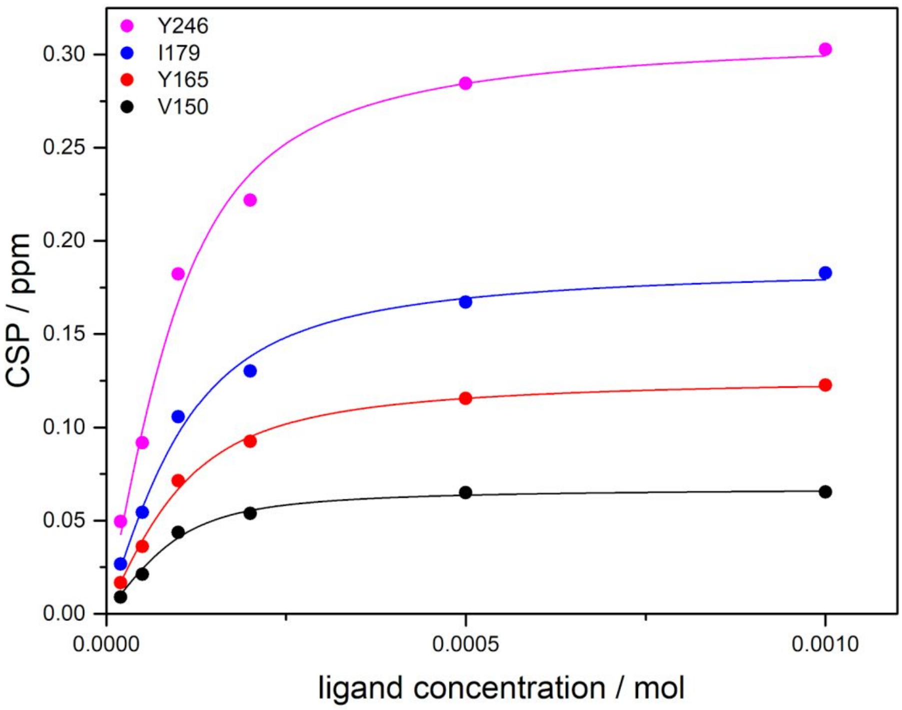


**Figure S7.** Selected titration curves from $^{1}H$-$^{15}N$ HSQC NMR spectra for $^{15}N$-labelled OPN titrated with heparin for the residues V150 (black), Y165 (red), I179 (blue) and Y246 (magenta), together with the corresponding fit for a fast chemical exchange (thin lines).

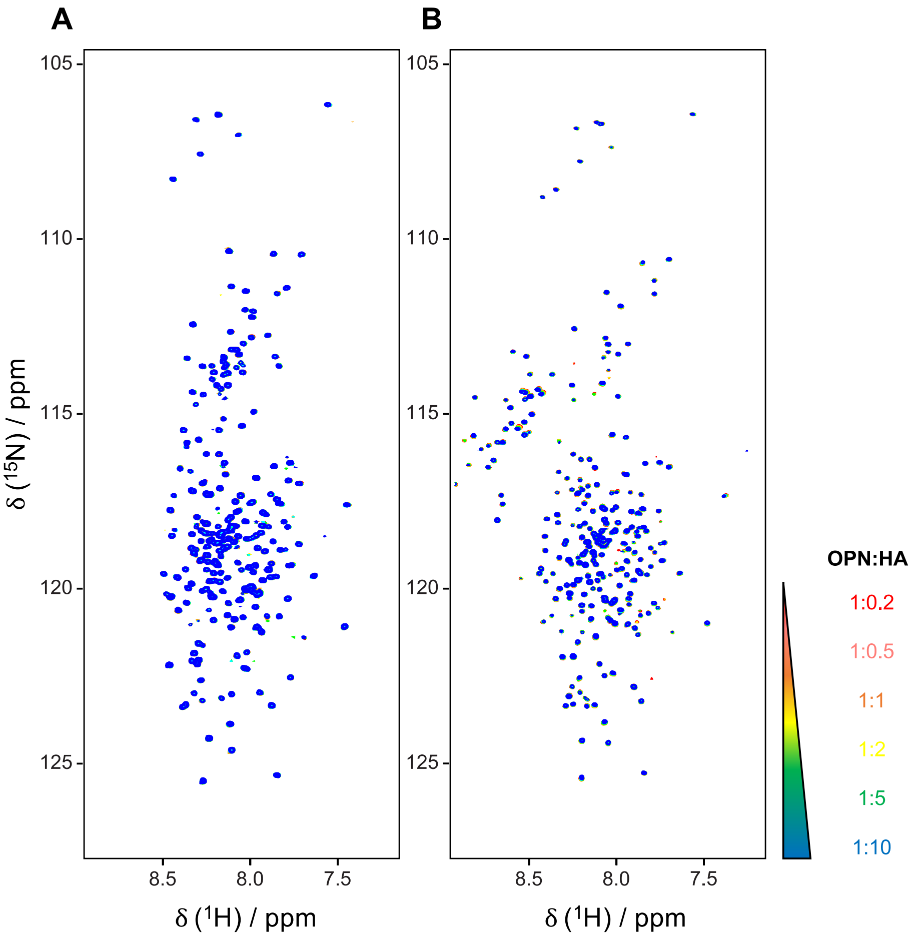


**Figure S8.** Series of $^{1}H$-$^{15}N$ HSQC NMR spectra ($B_0$ = 18.8 T, 293K) at different concentrations of hyaluronic acid (HA) binding to OPN (A) and phosphorylated OPN (B). The OPN concentration was 0.1mM and the molar ratio of OPN:HA was increased from 1:0.2 to 1:10.

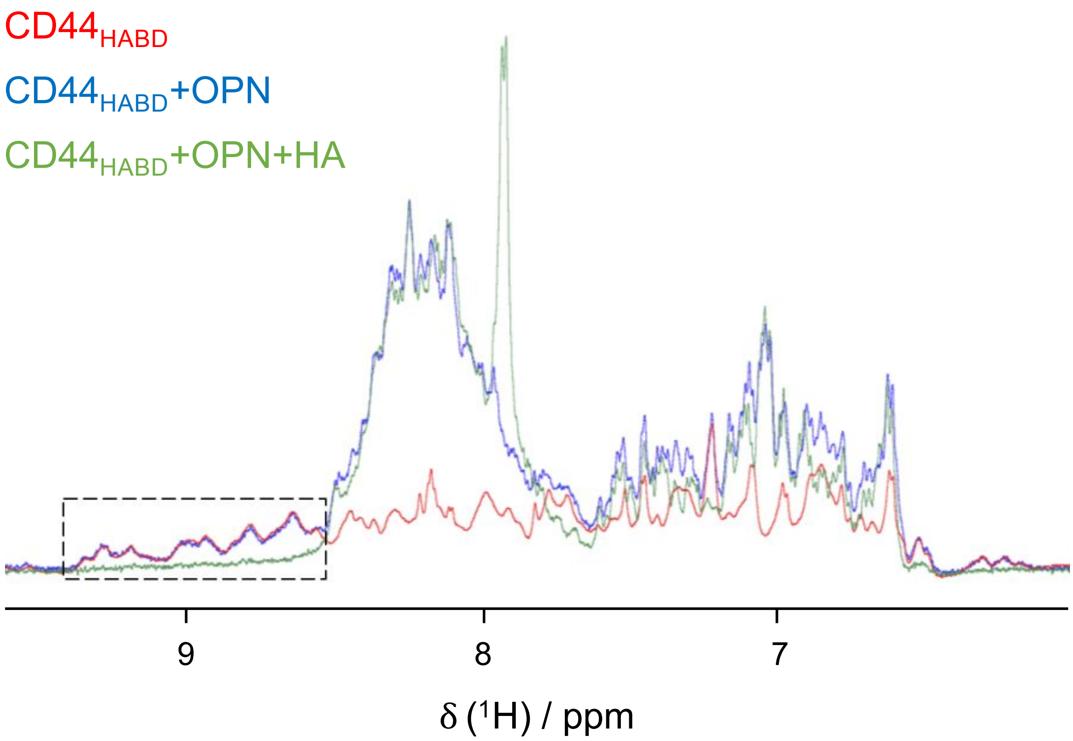


**Figure S9.** $^{1}H$ NMR spectra of Homo sapiens OPN (blue), OPN+$CD44_{HABD}$ (red) and OPN+$CD44_{HABD}$+HA (green). The dashed square in the region around 9 ppm shows functionality of $CD44_{HABD}$ binding to HA, resulting in an extensive line-broadening.

**Table S1**. Backbone chemical shifts of the phosphorylated *Homo sapiens* OPN recorded at T=293K on a Bruker Avance III 800 MHz spectrometer equipped with a standard PA-TXI probe.

| | $H^N$ (ppm) | C´ (ppm) | N (ppm) | $C^{\alpha}$ (ppm) | $C^{\beta}$ (ppm) |
|---|---|---|---|---|---|
| **A14** | | | | 52.58 | 19.25 |
| **M15** | 8.48 | 178.2 | 120.58 | 55.58 | 32.77 |
| **G16** | 8.37 | 175.1 | 110.93 | 45.14 | |
| **I17** | 7.92 | 176.19 | 122.49 | 58.7 | 38.45 |
| **V18** | 8.18 | 177.6 | 121.63 | 62.32 | 33.17 |
| **K20** | | 177.75 | 126.34 | 56.29 | 33.17 |
| **Q21** | 8.52 | 177.21 | 124.08 | 55.74 | 29.55 |
| **A22** | 8.42 | 178.92 | 126.39 | 52.76 | 19.29 |
| **D23** | 8.37 | 177.74 | 120.07 | 54.4 | 41.09 |
| **S24** | 8.24 | 176.62 | 116.6 | 58.62 | 64.11 |
| **G25** | 8.56 | 176.19 | 111.94 | 45.37 | |
| **S26** | 8.76 | 176.61 | 117.79 | 58.26 | 66.04 |
| **S27** | 8.88 | 176.38 | 119.24 | 58.71 | 65.77 |
| **E28** | 8.32 | 178.58 | 123.32 | 57.62 | |
| **E29** | 8.25 | 178.53 | 121.91 | 57.56 | 29.57 |
| **K30** | 8.11 | 178.53 | 121.97 | 56.99 | 32.7 |
| **Q31** | 8.18 | 177.66 | 120.57 | 56.06 | 28.91 |
| **L32** | 8.04 | 178.75 | 122.82 | 55.73 | 42.26 |
| **Y33** | 7.89 | 176.97 | 119.75 | 57.71 | 38.46 |
| **N34** | 8.1 | 176.14 | 120.48 | 53.2 | 38.85 |
| **K35** | 7.94 | 177.24 | 121.96 | 56.47 | 33.06 |
| **Y36** | 8.09 | 175.5 | 121.55 | 55.53 | 38.17 |
| **P37** | | 178.26 | | 63.55 | 31.94 |
| **D38** | 8.27 | 177.59 | 120.25 | 54.45 | 41 |
| **A39** | 8.08 | 179.2 | 124.44 | 52.66 | 19.26 |
| **V40** | 7.93 | 177.56 | 119.64 | 62.5 | 32.64 |
| **A41** | 8.21 | 179.42 | 127.7 | 52.61 | 18.92 |
| **T42** | 7.89 | 175.91 | 113.75 | 62.38 | 69.72 |
| **W43** | 7.83 | 177.16 | 122.83 | 57.42 | 29.17 |
| **L44** | 7.69 | 177.58 | 124.28 | 54.79 | 42.49 |
| **N45** | 8.05 | | 121.11 | | |
| **P48** | | 179 | | 63.94 | 32.04 |
| **S49** | 8.4 | 176.42 | 115.81 | 59.58 | 63.49 |
| **Q50** | 7.92 | 177.51 | 121.9 | 55.82 | 28.97 |
| **K51** | 8.11 | 178.2 | 122.51 | 56.89 | 32.84 |
| **Q52** | 8.34 | 177.19 | 121.43 | 56.12 | 29.46 |
| **N53** | 8.41 | 176.58 | 120.26 | 53.32 | 38.6 |
| **L54** | | 178.65 | | 55.5 | 42.22 |
| **L55** | 8.05 | 178.11 | 122.71 | 54.79 | 42.25 |
| **A56** | 8.04 | 176.78 | 126.56 | 50.48 | 18.15 |
| **P57** | | 178.53 | | 63.25 | 31.94 |
| **Q58** | 8.48 | 177.2 | 121.19 | 55.8 | 29.56 |
| **N59** | | 176.07 | | 53.21 | 39.12 |
| **A60** | 8.27 | 178.9 | 125.4 | 52.48 | 19.42 |
| **V61** | 8.12 | 177.9 | 119.88 | 61.95 | 33.09 |
| **S62** | 8.8 | 175.94 | 121.38 | 57.88 | 66.13 |
| **S63** | 8.65 | 175.48 | 118.9 | 57.99 | 66.06 |
| **E64** | 8.31 | 177.93 | 123.1 | 56.76 | 30.18 |
| **E65** | | 178.19 | | 56.63 | 30.18 |
| **T66** | 8.22 | 175.83 | 116.1 | 61.95 | 69.84 |
| **N67** | 8.44 | 176.28 | 121.89 | 53.31 | 39.15 |
| **D68** | 8.25 | 177.37 | 121.6 | 54.53 | 40.93 |
| **F69** | 8.09 | 177.14 | 121.13 | 57.95 | 39.03 |
| **K70** | 8.05 | 177.49 | 124.02 | 56.21 | 32.97 |
| **Q71** | 8.27 | 177.34 | 122.51 | 55.82 | 29.51 |
| **Q72** | 8.49 | 177.91 | 123.59 | 56.58 | 30.46 |
| **T73** | 8.25 | 175.64 | 117.34 | 61.92 | 69.93 |
| **L74** | 8.36 | 176.58 | 127.73 | 53.18 | 41.66 |
| **P75** | | 178.3 | | 63.11 | 32.07 |
| **S76** | 8.41 | 176.26 | 117.36 | 58.14 | 63.92 |
| **K77** | 8.47 | 178.38 | 124.58 | 56.41 | 33.04 |
| **S78** | 8.93 | 175.72 | 118.84 | 58.27 | 65.78 |
| **N79** | 8.36 | 176.45 | 120.49 | 53.33 | 39.16 |
| **E80** | 8.18 | 177.95 | 121.57 | 56.72 | 30.29 |
| **S81** | 8.73 | 175.85 | 118.12 | 57.88 | 65.82 |
| **H82** | 8.36 | 175.56 | 120.42 | 55.65 | 29.44 |

| | | | | | |
|---|---|---|---|---|---|
| **D83** | 8.38 | 177.54 | 121.5 | 54.61 | 41.02 |
| **H84** | 8.48 | 176.21 | 119.9 | 55.81 | 28.87 |
| **M85** | | 177.5 | | 55.75 | 32.72 |
| **D86** | 8.34 | 177.38 | 122.09 | 54.51 | 41.2 |
| **D87** | 8.18 | 177.39 | 120.94 | 54.55 | 41.05 |
| **D95** | | 177.53 | | | |
| **H96** | 8.28 | 175.92 | 119.66 | 55.86 | 28.7 |
| **V97** | 8.14 | 177.18 | 123.85 | 62.36 | 32.86 |
| **D98** | | 178.15 | | 54.55 | 41.42 |
| **S99** | 8.86 | 176.09 | 119.57 | 58.28 | 66.11 |
| **Q100** | 8.44 | 177.29 | 122.22 | 55.75 | 29.5 |
| **D101** | 8.21 | 177.67 | 122.13 | 54.56 | 41.18 |
| **S102** | 8.19 | 175.79 | 116.83 | 58.14 | 63.86 |
| **I103** | 8.17 | 177.37 | 123.42 | 60.93 | 38.9 |
| **D104** | 8.38 | 177.72 | 125.89 | 54.15 | 41.5 |
| **S105** | 8.68 | 175.36 | 118.48 | 57.39 | 66.32 |
| **N106** | 8.55 | 176.26 | 121.69 | 53.41 | 39.52 |
| **D107** | 8.34 | 177.88 | 122.57 | 54.34 | 41.3 |
| **S108** | 8.56 | 175.37 | 117.55 | 57.57 | 66.34 |
| **D109** | 8.39 | 177.32 | 123.3 | 54.5 | 41.27 |
| **D110** | 8.24 | 177.52 | 122.02 | 54.23 | 41.05 |
| **V111** | 8.03 | 177.31 | 120.89 | 62.18 | 32.78 |
| **D112** | 8.4 | 177.36 | 125.27 | 54.3 | 41.27 |
| **D113** | 8.34 | 177.96 | 122.97 | 54.11 | 41.07 |
| **T114** | 8.15 | 175.9 | 115.16 | 62.13 | 70.01 |
| **D115** | 8.36 | 177.34 | 123.79 | 54.13 | 41.17 |
| **D116** | 8.25 | 177.92 | 122.31 | 54.36 | 41.4 |
| **S117** | 8.65 | 175.79 | 117.71 | 57.63 | 66.12 |
| **H118** | 8.48 | 175.6 | 121.39 | 56.24 | 28.94 |
| **Q119** | 8.42 | 177.35 | 123.58 | 55.57 | 29.66 |
| **S120** | 8.82 | 175.21 | 119.57 | 57.81 | 66.05 |
| **D121** | 8.47 | 177.5 | 123.31 | 54.51 | 41.13 |
| **E122** | 8.25 | 177.75 | 121.84 | 56.33 | 30.72 |
| **S123** | 8.66 | 175.18 | 118.44 | 57.15 | 66.16 |
| **H124** | 8.54 | 175.44 | 121.27 | 55.78 | 29.55 |
| **H125** | 8.66 | 175.79 | 123.04 | 54.73 | 29.61 |
| **S126** | 8.96 | 175.15 | 119.69 | 58.1 | 65.86 |
| **D127** | 8.58 | 177.6 | 122.91 | 54.47 | 41.03 |
| **E128** | 8.29 | 178.14 | 121.85 | 56.64 | 30.35 |
| **S129** | 8.61 | 175.33 | 118.24 | 57.65 | 66.31 |
| **D130** | 8.42 | 177.6 | 123.4 | 54.5 | 41.32 |
| **E131** | 8.3 | 177.71 | 122.25 | 56.42 | 30.18 |
| **L132** | 8.28 | 178.65 | 124.77 | 55.2 | 42.13 |
| **V133** | 8.19 | 177.73 | 123.7 | 62.39 | 32.65 |
| **T134** | 8.19 | 175.22 | 118.81 | 61.42 | 69.95 |
| **D135** | 8.1 | 176.63 | 123.42 | 54.1 | 41.36 |
| **F136** | 8.11 | 175.34 | 122.17 | 55.6 | 39.02 |
| **P137** | | 178.54 | | 63.23 | 32.04 |
| **T138** | 8.22 | 175.74 | 114.7 | 61.8 | 69.97 |
| **D139** | 8.27 | 177.13 | 123.08 | 54.02 | 41.12 |
| **L140** | 8.12 | 176.63 | 124.38 | 53.12 | 41.65 |
| **P141** | | 178.17 | | 63.01 | 33.07 |
| **A142** | 8.4 | 179.54 | 125.28 | 52.63 | 19.16 |
| **T143** | 8.04 | 175.95 | 113.91 | 61.99 | 69.82 |
| **E144** | 8.31 | 177.38 | 124.1 | 56.44 | 30.48 |
| **V145** | 8.08 | 177.08 | 122.24 | 62.13 | 32.85 |
| **F146** | 8.38 | 176.71 | 125.74 | 57.62 | 39.77 |
| **T147** | 8.06 | 173.4 | 121.54 | 59.41 | 70.05 |
| **P148** | | 177.33 | | | |
| **V149** | 8.23 | 177.67 | 121.9 | 62.43 | 32.68 |
| **V150** | 8.24 | 175.81 | 127.1 | 60.02 | 32.36 |
| **P151** | | 178.38 | | 63.23 | 32.09 |
| **T152** | 8.23 | 176.11 | 115.98 | 61.87 | 70.05 |
| **V153** | 8.14 | 177.1 | 122.59 | 62.05 | 32.96 |
| **D154** | 8.38 | 177.73 | 124.8 | 54.48 | 41.34 |
| **T155** | 7.97 | 175.84 | 114.75 | 61.72 | 69.82 |
| **Y156** | 8.25 | 177.11 | 123.26 | 58.24 | 38.65 |
| **D157** | 8.18 | 178.13 | 123.6 | 54.02 | 41.12 |
| **G158** | 7.77 | 176.16 | 109.57 | 45.77 | |
| **R159** | 8.03 | 178.61 | 120.53 | 56.67 | 30.67 |
| **G160** | 8.39 | 175.5 | 110 | 45.47 | |
| **D161** | 8.19 | 177.98 | 121.02 | 54.48 | 41.08 |

| | | | | | |
|---|---|---|---|---|---|
| **S162** | 8.22 | 176.24 | 116.48 | 58.83 | 63.72 |
| **V163** | 8.01 | 177.59 | 122.95 | 62.93 | 32.42 |
| **V164** | 7.97 | 177.5 | 123.72 | 62.65 | 32.76 |
| **Y165** | 8.18 | 178.05 | 124.33 | 58.63 | 38.86 |
| **G166** | 8.2 | 175.56 | 110.51 | 45.42 | |
| **L167** | 7.98 | 179.19 | 122.12 | 55.49 | 42.51 |
| **R168** | | 177.91 | | | |
| **S169** | 8.24 | 175.64 | 117.2 | | |
| **S171** | 8.21 | | 117.01 | | |
| **I179** | 7.68 | 176.87 | 120.86 | 60.8 | 39.07 |
| **Q180** | 8.22 | | 124.17 | | |
| **D183** | | 177.23 | | 54.2 | 40.93 |
| **A184** | 8.05 | 179.16 | 124.68 | 52.38 | 19.66 |
| **T185** | 8.72 | 175.41 | 116.43 | 62.35 | 73.2 |
| **E187** | | 177.55 | | 56.66 | 30.39 |
| **D188** | 8.48 | 177.59 | 122.38 | 54.46 | 41 |
| **I189** | 8.08 | 177.91 | 121.76 | 61.13 | 38.54 |
| **T190** | 8.12 | 175.92 | 118.94 | 61.76 | 70.13 |
| **S191** | 8.8 | 175.64 | 119.12 | 57.95 | 66.15 |
| **H192** | 8.32 | | 120.43 | 55.73 | 29.06 |
| **M193** | | 177.76 | | 55.84 | 33.08 |
| **E194** | 8.57 | 178.38 | 122.75 | 56.96 | 30.13 |
| **S195** | 8.73 | 176.03 | 118.5 | 58.32 | 65.91 |
| **E196** | 8.43 | 178.15 | 123.34 | 57.04 | 30.31 |
| **E197** | 8.29 | 178.36 | 122.36 | 57.12 | 30.08 |
| **L198** | 8.23 | 179.05 | 123.58 | 55.69 | 42.12 |
| **N199** | 8.39 | 177.46 | 119.47 | 53.73 | 38.87 |
| **G200** | 8.28 | 175.62 | 109.81 | 45.75 | |
| **A201** | 8.01 | 179.05 | 123.94 | 52.67 | 19 |
| **Y202** | 8.02 | 177.08 | 119.66 | 58.08 | 38.57 |
| **K203** | 7.89 | 176.81 | 124.55 | 55.81 | 33.29 |
| **A204** | 8.07 | 178.79 | 126.09 | 52.1 | 19.07 |
| **I205** | 8.08 | 176.14 | 123.06 | 58.71 | 38.53 |
| **P206** | | 178.19 | | 63.1 | 32.1 |
| **V207** | 8.17 | 177.52 | 121.43 | 62.21 | 32.89 |
| **A208** | 8.36 | 179.03 | 128.66 | 52.58 | 19.22 |
| **Q209** | 8.32 | 176.98 | 120.58 | 55.82 | 29.75 |
| **D210** | 8.35 | 177.86 | 122.3 | 54.46 | 41.01 |
| **L211** | 8.22 | 178.77 | 123.83 | 55.51 | 42.05 |
| **N212** | 8.36 | 175.91 | 119.26 | 53.21 | 38.98 |
| **A213** | 7.98 | 176.78 | 125.88 | 50.72 | 18.23 |
| **P214** | | 178.6 | | 63.21 | 32.09 |
| **S215** | 8.39 | 175.9 | 116.84 | 58.37 | 63.89 |
| **D216** | 8.26 | 177.27 | 122.59 | 54.48 | 41.05 |
| **W217** | 7.94 | 177.07 | 121.41 | 57.19 | 29.7 |
| **D218** | 7.92 | 177.59 | 123.58 | 53.89 | 41.46 |
| **S219** | 8.68 | 176.36 | 118.73 | 58.22 | 65.92 |
| **R220** | 8.25 | 178.62 | 122.74 | 56.62 | 30.29 |
| **G221** | 8.27 | 175.65 | 109.85 | 45.51 | |
| **K222** | 8.06 | 177.9 | 121.13 | 56.19 | 33.15 |
| **D223** | 8.34 | 177.85 | 122.26 | 54.48 | 41.37 |
| **S224** | 8.51 | 175.24 | 117.07 | 57.63 | 66.13 |
| **Y225** | 8.11 | 176.88 | 122.33 | | |
| **E226** | | 177.56 | | 56.25 | 30.42 |
| **T227** | 8.12 | 175.95 | 116.24 | 61.87 | 69.9 |
| **S228** | 8.86 | 175.82 | 119.78 | 57.98 | 66.03 |
| **Q229** | 8.29 | 177.15 | 122.48 | 55.54 | 29.29 |
| **Q233** | | 178.46 | | 56.42 | 32.81 |
| **S234** | 8.77 | 175.96 | 118.83 | 57.99 | 65.85 |
| **A235** | 8.24 | 179.82 | 125.72 | 53.37 | 19.21 |
| **E236** | 8.31 | 178.67 | 119.52 | 57.29 | 29.85 |
| **T237** | 7.97 | 176.4 | 114.33 | 62.64 | 69.54 |
| **S243** | | 175.75 | | 58.62 | 64 |
| **R244** | 8.34 | 177.66 | 123.43 | 56.54 | 30.24 |
| **L245** | 8.04 | 178.45 | 122.47 | 55.33 | 42.32 |
| **Y246** | 7.95 | 176.89 | 121.01 | 57.81 | 38.9 |
| **K247** | 8.04 | 177.37 | 123.98 | | |
| **R248** | | 177.7 | | | 30.83 |
| **K249** | 8.39 | 177.79 | 124.06 | 56.38 | 33.15 |
| **A250** | 8.4 | 179.02 | 126.17 | 52.69 | 19.31 |
| **N251** | 8.41 | 176.46 | 117.79 | 53.3 | 38.82 |
| **D252** | 8.21 | | 121.1 | | |

| | | | | | |
|---|---|---|---|---|---|
| **E253** | | 178.22 | | 56.68 | 29.48 |
| **S254** | 8.65 | 175.72 | 117.94 | 57.97 | 65.83 |
| **N255** | 8.38 | 176.54 | 121.04 | 53.44 | 39.38 |
| **E256** | 8.33 | 177.75 | 121.39 | 56.93 | 29.98 |
| **H257** | 8.48 | 176.03 | 120.22 | 55.36 | 29.26 |
| **S258** | 8.33 | | 118.04 | | |
| **D259** | | 177.35 | | 54.16 | 40.93 |
| **V260** | 8.02 | 177.37 | 121 | 62.33 | 32.7 |
| **I261** | 8.24 | 177.12 | 126.41 | 60.8 | 38.87 |
| **D262** | 8.39 | 178.15 | 126.51 | 53.98 | 41.69 |
| **S263** | 9.03 | 176.74 | 120.21 | 58.91 | 65.74 |
| **Q264** | 8.43 | 177.95 | 121.97 | 56.35 | 28.94 |
| **E265** | 8.01 | 178.39 | 121.62 | 57.04 | 29.89 |
| **L266** | 8.09 | 179.22 | 123 | 55.71 | 42.14 |
| **S267** | 8.16 | 176.04 | 116.49 | 58.64 | 63.65 |
| **K268** | 8.15 | 178.06 | 124.02 | 56.46 | 33 |
| **V269** | 8.03 | 177.94 | 121.52 | 62.33 | 32.83 |
| **S270** | 8.78 | 175.88 | 120.84 | 58.04 | 65.84 |
| **S275** | 9.1 | | 119.1 | | |
| **H279** | | | | | 29.67 |
| **S280** | 9.08 | | 119.1 | 58.39 | 65.83 |
| **H281** | 8.37 | | 119.53 | 55.77 | 29.48 |
| **M284** | | 177.36 | | 55.44 | |
| **L285** | 8.21 | 178.43 | 124.12 | 55.2 | 42.29 |
| **V286** | 8.13 | 177.32 | 123.32 | 62.4 | 32.67 |
| **V287** | 8.17 | 176.9 | 125.61 | 61.94 | 33.16 |
| **D288** | 8.44 | | 126.63 | 52.26 | 40.9 |
| **D295** | | | | | 40.86 |
| **D307** | | 177.86 | | | |
| **S308** | 8.62 | 175.6 | 117.75 | 57.65 | 66.15 |
| **A309** | 8.31 | 179.55 | 126.57 | 52.77 | 19.45 |
| **S310** | 8.65 | 175.85 | 116.53 | 57.91 | 65.93 |
| **S311** | 8.16 | 175.79 | 117.78 | 58.87 | 64.13 |
| **E312** | 8.2 | 177.68 | 123.24 | 56.63 | 30.21 |
| **V313** | 8.08 | 176.62 | 121.71 | 62.33 | 32.73 |
| **N314** | 8.03 | | 128.56 | 54.84 | 40.68 |

**Table S2.** Acquisition parameters of the $^{1}H$ detected experiments for phosphorylated OPN assignment with at 293K.

| | Dimension of acquired data | | | Spectral width (ppm) | | | n[a] | d[b] |
|---|---|---|---|---|---|---|---|---|
| | $t_1$ | $t_2$ | $t_3$ | $F_1$ | $F_2$ | $F_3$ | | |
| BT-HSQC | 768 ($^{15}N$) | 2048 ($^{1}H$) | | 35 | 16 | | 8 | 0.2 |
| BT-HNCACB | 128 ($^{13}C$) | 96 ($^{15}N$) | 1536 ($^{1}H$) | 80 | 25 | 14 | 8 | 0.2 |
| BT-HN(CO)CACB | 128 ($^{13}C$) | 96 ($^{15}N$) | 1536 ($^{1}H$) | 80 | 25 | 14 | 8 | 0.2 |
| BT-HN(CA)NNH | 116 ($^{13}C^{\alpha}$) | 116 ($^{15}N$) | 1536 ($^{1}H$) | 25 | 25 | 14 | 8 | 0.2 |
| BT- HN(COCA)NNH | 116 ($^{13}C$) | 116 ($^{15}N$) | 1536 ($^{1}H$) | 25 | 25 | 14 | 8 | 0.2 |
| BT-HNCO | 128 ($^{13}C$) | 96 ($^{15}N$) | 1536 ($^{1}H$) | 10 | 25 | 14 | 4 | 0.1 |
| BT-HN(CA)CO | 128 ($^{13}C$) | 96 ($^{15}N$) | 1536 ($^{1}H$) | 10 | 25 | 14 | 8 | 0.2 |

[a] Number of acquired scans.
[b] Relaxation delay in seconds.

**Table S3.** Acquisition parameters of the $^{1}H$ detected PRE and relaxation experiments.

| | Dimension of acquired data | | | Spectral width (ppm) | | | n[a] | d[b] |
|---|---|---|---|---|---|---|---|---|
| | $t_1$ | $t_2$ | $t_3$ | $F_1$ | $F_2$ | $F_3$ | | |
| $^{1}H$-$T_2$ PRE qOPN | 768 ($^{15}N$) | 2048 ($^{1}H$) | | 32 | 14 | | 4 | 1.5 |
| $^{1}H$-$T_2$ PRE hOPN | 512 ($^{15}N$) | 2048 ($^{1}H$) | | 32 | 14 | | 8 | 1.5 |
| $^{15}N$-$T_1$ | 640 ($^{15}N$) | 2048 ($^{1}H$) | | 32 | 16 | | 8 | 1.4 |
| $^{15}N$-$T_2$ | 640 ($^{15}N$) | 2048 ($^{1}H$) | | 32 | 16 | | 4 | 1.4 |
| $^{15}N$-{$^{1}H$} hetNOE | 256 ($^{15}N$) | 2048 ($^{1}H$) | | 34 | 16 | | 8 | 10 |

[a] Number of acquired scans.
[b] Relaxation delay in seconds.

## SI References


(1) Bracken, C.; Marley, J.; Lu, M. (2001) A Method for Efficient Isotopic Labeling of Recombinant Proteins. J Biomol NMR 20, 71–75.

(2) Tagliabracci, V. S.; Engel, J. L.; Wen, J.; Wiley, S. E.; Worby, C. a; Kinch, L. N.; Xiao, J.; Grishin, N. V; Dixon, J. E. (2012) Secreted Kinase Phosphorylates Extracellular Proteins That Regulate Biomineralization. Science 336, 1150–1153.

(3) Solyom, Z.; Schwarten, M.; Geist, L.; Konrat, R.; Willbold, D.; Brutscher, B. (2013) BEST-TROSY Experiments for Time-Efficient Sequential Resonance Assignment of Large Disordered Proteins. J. Biomol. NMR 55, 311–321.

(4) Vranken, W. F.; Boucher, W.; Stevens, T. J.; Fogh, R. H.; Pajon, A.; Llinas, M.; Ulrich, E. L.; Markley, J. L.; Ionides, J.; Laue, E. D. (2005) The CCPN Data Model for NMR Spectroscopy: Development of a Software Pipeline. Proteins Struct. Funct. Genet. 59, 687–696.

(5) Marsh, J. A.; Singh, V. K.; Jia, Z.; Forman-Kay, J. D. (2006) Sensitivity of Secondary Structure Propensities to Sequence Differences between α- and γ-Synuclein: Implications for Fibrillation. Protein Sci. 15, 2795–2804.

(6) Nielsen, J. T.; Mulder, F. A. A. (2018) POTENCI: Prediction of Temperature, Neighbor and PH-Corrected Chemical Shifts for Intrinsically Disordered Proteins. J. Biomol. NMR 70, 141-165.

(7) Ferrage, F.; Piserchio, A.; Cowburn, D.; Ghose, R. (2008) On the Measurement of $^{15}$N-{$^{1}$H} Nuclear Overhauser Effects. J. Magn. Reson. 192, 302–313.

(8) Rappsilber, J.; Mann, M.; Ishihama, Y. (2007) Protocol for Micro-Purification, Enrichment, Pre-Fractionation and Storage of Peptides for Proteomics Using StageTips. Nat. Protoc. 2, 1896–1906.

(9) Cox, J.; Mann, M. (2008) MaxQuant Enables High Peptide Identification Rates, Individualized p.p.b.-Range Mass Accuracies and Proteome-Wide Protein Quantification. Nat. Biotechnol. 26, 1367–1372.

(10) Christensen, B.; Nielsen, M. S.; Haselmann, K. F.; Petersen, T. E.; Sørensen, E. S. (2005) Post-Translationally Modified Residues of Native Human Osteopontin Are Located in Clusters: Identification of 36 Phosphorylation and Five O-Glycosylation Sites and Their Biological Implications. Biochem. J.390, 285–292.

(11) Tagliabracci, V. S.; Engel, J. L.; Wen, J.; Wiley, S. E.; Worby, C. A.; Kinch, L. N.; Xiao, J.; Grishin, N. V; Dixon, J. E. (2012) Secreted Kinase Phosphorylates Extracellular Proteins That Regulate Biomineralization. Science 336, 1150–1153.

(12) Smith, L. J.; Fiebig, K. M.; Schwalbe, H.; Dobson, C. M. (1996) The Concept of a Random Coil. Residual Structure in Peptides and Denatured Proteins. Fold. Des. 1, R95–R106.

(13) Platzer, G.; Żerko, S.; Saxena, S.; Koźmiński, W.; Konrat, R. (2015) $^{1}$H, $^{15}$N, $^{13}$C Resonance Assignment of Human Osteopontin. Biomol. NMR Assign. 9, 289-292.

(14) Conibear, A. C.; Rosengren, K. J.; Becker, C. F. W.; Kaehlig, H. (2019) Random Coil Shifts of Posttranslationally Modified Amino Acids. J. Biomol. NMR 73, 587–599.

(15) Hendus-Altenburger, R.; Fernandes, C. B.; Bugge, K.; Kunze, M. B. A.;

Boomsma, W.; Kragelund, B. B. Random (2019) Coil Chemical Shifts for Serine, Threonine and Tyrosine Phosphorylation over a Broad PH Range. J. Biomol. NMR 73, 713–725.
